# A Systems Biology Approach Reveals that Tissue Tropism to West Nile Virus Is Regulated by Antiviral Genes and Innate Immune Cellular Processes

**DOI:** 10.1371/journal.ppat.1003168

**Published:** 2013-02-07

**Authors:** Mehul S. Suthar, Margaret M. Brassil, Gabriele Blahnik, Aimee McMillan, Hilario J. Ramos, Sean C. Proll, Sarah E. Belisle, Michael G. Katze, Michael Gale

**Affiliations:** 1 Department of Immunology, University of Washington School of Medicine, Seattle, Washington, United States of America; 2 Department of Microbiology, University of Washington School of Medicine, Seattle, Washington, United States of America; Harvard Medical School, United States of America

## Abstract

The actions of the RIG-I like receptor (RLR) and type I interferon (IFN) signaling pathways are essential for a protective innate immune response against the emerging flavivirus West Nile virus (WNV). In mice lacking RLR or IFN signaling pathways, WNV exhibits enhanced tissue tropism, indicating that specific host factors of innate immune defense restrict WNV infection and dissemination in peripheral tissues. However, the immune mechanisms by which the RLR and IFN pathways coordinate and function to impart restriction of WNV infection are not well defined. Using a systems biology approach, we defined the host innate immune response signature and actions that restrict WNV tissue tropism. Transcriptional profiling and pathway modeling to compare WNV-infected permissive (spleen) and nonpermissive (liver) tissues showed high enrichment for inflammatory responses, including pattern recognition receptors and IFN signaling pathways, that define restriction of WNV replication in the liver. Assessment of infected livers from *Mavs^−/−^×Ifnar^−/−^* mice revealed the loss of expression of several key components within the natural killer (NK) cell signaling pathway, including genes associated with NK cell activation, inflammatory cytokine production, and NK cell receptor signaling. *In vivo* analysis of hepatic immune cell infiltrates from WT mice demonstrated that WNV infection leads to an increase in NK cell numbers with enhanced proliferation, maturation, and effector action. In contrast, livers from *Mavs^−/−^×Ifnar^−/−^* infected mice displayed reduced immune cell infiltration, including a significant reduction in NK cell numbers. Analysis of cocultures of dendritic and NK cells revealed both cell-intrinsic and -extrinsic roles for the RLR and IFN signaling pathways to regulate NK cell effector activity. Taken together, these observations reveal a complex innate immune signaling network, regulated by the RLR and IFN signaling pathways, that drives tissue-specific antiviral effector gene expression and innate immune cellular processes that control tissue tropism to WNV infection.

## Author Summary

West Nile virus (WNV), a mosquito-transmitted RNA flavivirus, is an NIAID Category B infectious agent that has emerged in the Western hemisphere as a serious public health threat. The innate immune effectors that impart restriction of WNV infection are not well defined. WNV infection is sensed by the host RIG-I like receptors (RLR), a class of pattern recognition receptors, to trigger type I interferon (IFN) and related innate immune defense programs. Using a systems biology approach, we evaluated the contribution of the RLR and type I IFN signaling pathways in controlling tissue tropism. WNV infection triggers tissue-specific innate immune responses, specifically antiviral effector genes and natural killer (NK) cell signaling related genes, which are directly regulated by the combined actions of the RLR and type I IFN signaling pathways. Cocultures of dendritic and NK cells revealed that RLR and type I IFN signaling pathways are essential in promoting NK cell activation during WNV infection. Our observations indicate that combined RLR- and type I IFN-dependent signaling programs drive specific antiviral effector gene expression and programs NK cell responses that, together, serve to restrict WNV tissue tropism.

## Introduction

Acute virus infection induces host innate immune defense programs that serve to control virus replication, prevent virus-mediated pathology, and aid in developing sterilizing immunity (i.e. humoral and cell-mediated immunity). During RNA virus infection, intracellular viral RNA is recognized as a non-self pathogen associated molecular pattern (PAMP) by the RIG-I like receptors (RLR), RIG-I and MDA5 [1], [2]. Upon binding virus-specific RNA structures and target nucleic acid sequences, RIG-I and MDA5 undergo conformational change and interact with the adaptor protein MAVS, leading to activation of NF-κB and interferon regulatory factor (IRF), including IRF-3 and IRF-7, that drive transcription of antiviral target genes, interferon-β, pro-inflammatory cytokines, and hundreds of interferon-stimulated genes (ISGs) [1], [3]. This host response is further amplified by signaling through the type I interferon (IFN) receptor that drives the formation of the multimeric interferon-stimulated gene factor 3 (ISGF3), consisting of IRF-9, STAT2 and/or STAT1, that binds to interferon-stimulated response promoter elements (ISRE) and further amplifies the transcription of ISGs. While several studies have identified key innate immune host factors in controlling virus replication and protection, little is known about context (specific cell types and organs) and how these processes regulate innate immune responses to restrict tropism of virus infection

West Nile virus (WNV) is an emerging neurotropic flavivirus that is the leading cause of mosquito-borne encephalitis in humans in the United States. The WNV pathogenesis model of infection in mice provides a platform to study immune response processes and pathways that regulate infection. After subcutaneous footpad inoculation in mice, WNV initially replicates in the skin at the inoculation site and the draining popliteal lymph node, resulting in a primary viremia and spread to the spleen. Within the spleen the virus is amplified and following a secondary viremia, WNV invades the central nervous system tissues (e.g., brain and spinal cord), thus recapitulating infection and pathogenesis of human infection transmitted by mosquito vector [4]. WNV replication is typically restricted to the skin, draining lymph node, spleen, and central nervous system in humans and WT mice [4], [5]. Low levels of infectious virus can be recovered from the lung, kidney, heart, pancreas and other peripheral tissues but not the liver, of WT infected mice [6]. Most peripheral organs, including the liver, are not typically associated with WNV replication in humans. However, reported cases of kidney, liver, and heart organ transplant-transmitted WNV infections have been described with outcomes ranging from asymptomatic infections to death in the recipients [7]. These clinical observations suggest that peripheral organs in humans are also capable of being infected by WNV but infection is restricted or controlled by immune defense programs.

The RLR and the type I IFN signaling pathways are essential in eliciting innate immune responses that restrict WNV infection, control tissue tropism, and serve to program downstream adaptive immune responses [8]–[11]. During WNV infection, RIG-I is essential for triggering early antiviral immune defenses, whereas MDA5 serves to enhance and sustain this response [12]. Known target cells of WNV infection, including dendritic cells, macrophages, and neurons rely exclusively on RLR signaling to control WNV replication and drive innate antiviral immune defenses [8]. In the absence of the RLR or type I IFN signaling, WNV displays enhanced virus replication and tissue tropism [8], [13], indicating that the RLR and type I IFN signaling pathways impart a host response that restricts tissue permissiveness to WNV. Recent studies have identified several antiviral effector genes that control specific stages in the life cycle of WNV infection. Most notably, the interferon-induced protein with tetratricopeptide repeats (IFIT) family members [14]–[16], interferon induced transmembrane proteins (IFITM) 1 and 2 [17], [18], radical S-adenosyl methionine domain containing 2 (RSAD2; commonly known as viperin; [19], ISG20 [17], and interferon-inducible double stranded RNA activated kinase (PKR; [20]) have been shown to directly inhibit WNV infection. In addition, a high-throughput antiviral effector gene screen revealed hundreds of ISGs whose expression corresponds with IFN-mediated restriction of WNV replication, however, the biological significance of these genes in regulating pathogenesis is not defined [21]. Moreover, little is known of how these ISGs function in a context-dependent (e.g. in specific cells and tissues) manner to limit virus replication and spread *in vivo*.

The production of and response to type I IFN during the acute stage of virus infection is a major linkage point between innate and adaptive immunity. IFN-α and IFN-β have been shown to sustain B cell activation and differentiation [22]–[24], support the expansion of antigen-specific CD4^+^ and CD8^+^ T cells [25]–[28], and enhance the activation of natural killer (NK) cell responses against infection [29]. Moreover, recent studies have implicated the RLR signaling pathway in regulating inflammation, humoral immune responses, and T cell responses during viral infection [8]. Specifically, the absence of MAVS leads to excessive inflammation characterized by enhanced pro-inflammatory cytokine and chemokine production, enhanced virus-specific T cell responses, dysregulated humoral responses, and defective expansion of regulatory T cells during WNV infection [8]. In addition, the RLR LGP2 was found to be essential in regulating T cell survival and effector functions during virus infection [30]. These findings implicate the RLRs and their signaling as well as the induction of the IFN response as integral processes that restrict infection by containing acute virus replication and spread while bridging innate and adaptive immune responses to systemically control infection.

Here, we interrogated WNV infection in mice utilizing a systems biology approach comprising of genomics analyses, immunologic assessments, computational modeling, and hypothesis testing to define the nature of a protective innate immune response in controlling flavivirus tissue tropism and restriction of acute infection. We directly compared *in vivo* innate immune signaling and effector functions within the spleen, a permissive tissue for WNV infection, and the liver, a nonpermissive tissue to WNV infection, among WT and innate immune-deficient mouse lines. Our results define RLR and type I IFN signaling programs as pivotal immunologic regulatory nodes of WNV restriction. In addition, these studies demonstrate that hepatic tropism of WNV infection is controlled through a novel axis of RLR and type I IFN signaling linked to antiviral and innate immune cellular signaling pathways.

## Results

### Combinatorial actions of RLR and type I IFN signaling pathways are required for protection against WNV infection

**Figure 1:**
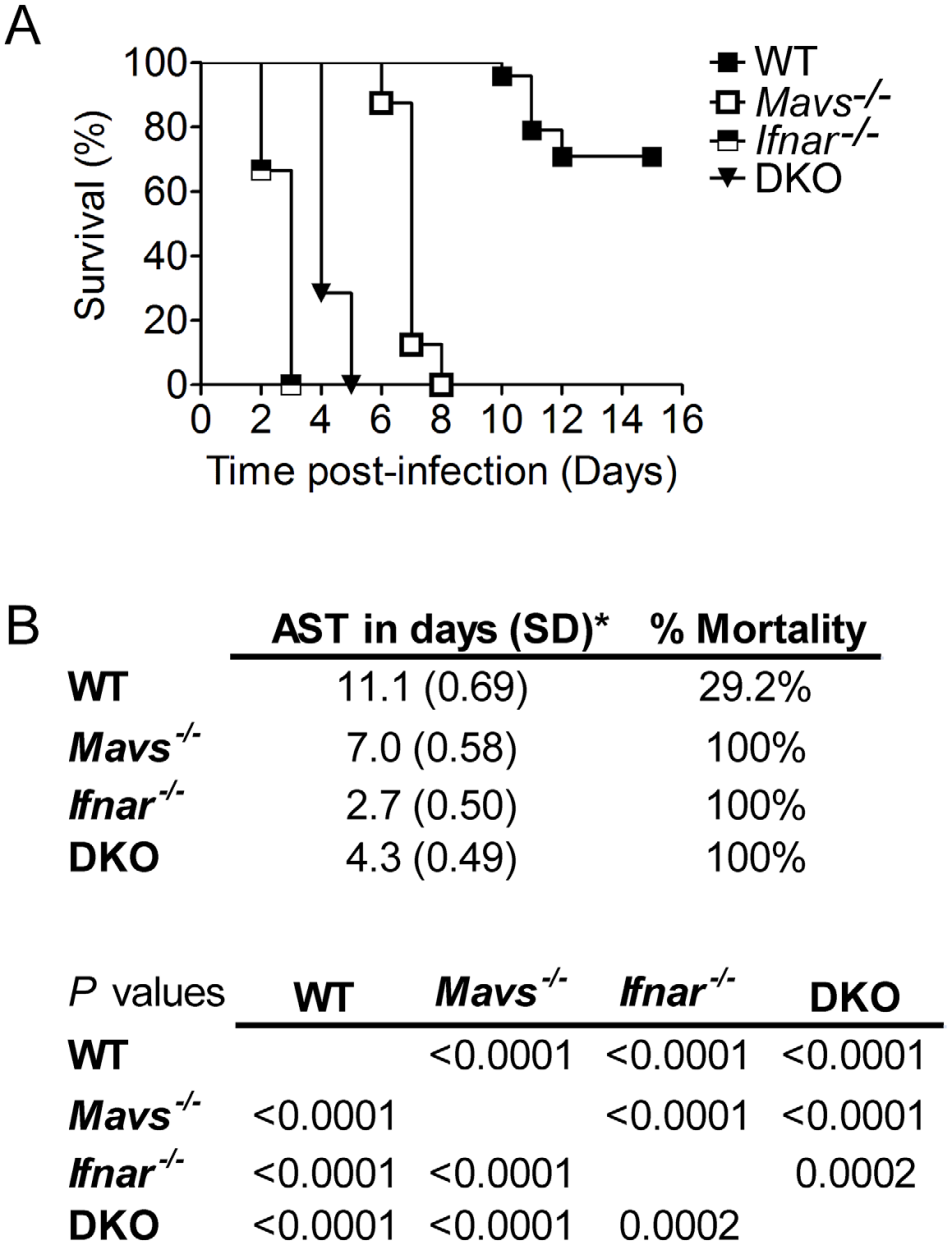
Enhanced virulence in mice lacking RLR and type I IFN signaling. (**A**) Survival of WT (closed squares; n = 24), *Mavs^−/−^* (open squares; n = 8), *Ifnar^−/−^* (half-closed square; n = 9), and *Mavs^−/−^*×*Ifnar^−/−^* (DKO; closed triangle; n = 7) adult mice infected subcutaneously with 100 PFU of WNV-TX. (**B**) Upper table represents average survival time (AST), standard deviation (SD), and percent mortality. Lower table represents *P* values comparing WT and KO mice infected with WNV-TX.

To evaluate the roles of the RLR and type I IFN signaling pathways in protection against WNV infection, wild-type (C57BL/6), *Mavs* ^−/−^, *Ifnar^−/−^*, and *Mavs^−/−^*×*Ifnar^−/−^* (herein referred to as DKO) mice were challenged with WNV-TX, a virulent lineage I and emergent West Nile virus strain (Figure 1A). All mouse lines were derived directly in pure C57BL/6 genetic background or were fully backcrossed to derive a pure C57BL/6 strain. *Mavs* ^−/−^ mice displayed a significant increase in mortality (from 29.2% to 100% mortality; *P*<0.0001) as compared to WT infected mice, demonstrating the importance of the RLR signaling pathway in protection against WNV infection (Figure 1B; [8]). We also observed a significant increase in mortality of *Ifnar* ^−/−^ mice from WNV infection (from 29.2% to 100% mortality; *P*<0.0001), with mice succumbing to WNV within 2.7 days post infection (p.i.), consistent with other studies demonstrating the importance of type I IFN signaling in protection against WNV infection [31]. DKO mice also displayed a significant increase in susceptibility to WNV infection over WT mice (100% mortality compared to 29% mortality, *P*<0.0001). Remarkably, we observed a significant increase in average survival time of WNV-infected DKO mice as compared to *Ifnar* ^−/−^ infected mice. Together, these findings demonstrate that the RLR and type I IFN signaling pathways mediate protection against WNV infection but may also serve to modulate the immune response to infection.

### Modulation of tissue tropism by the RLR signaling and type I IFN responses

**Figure 2:**
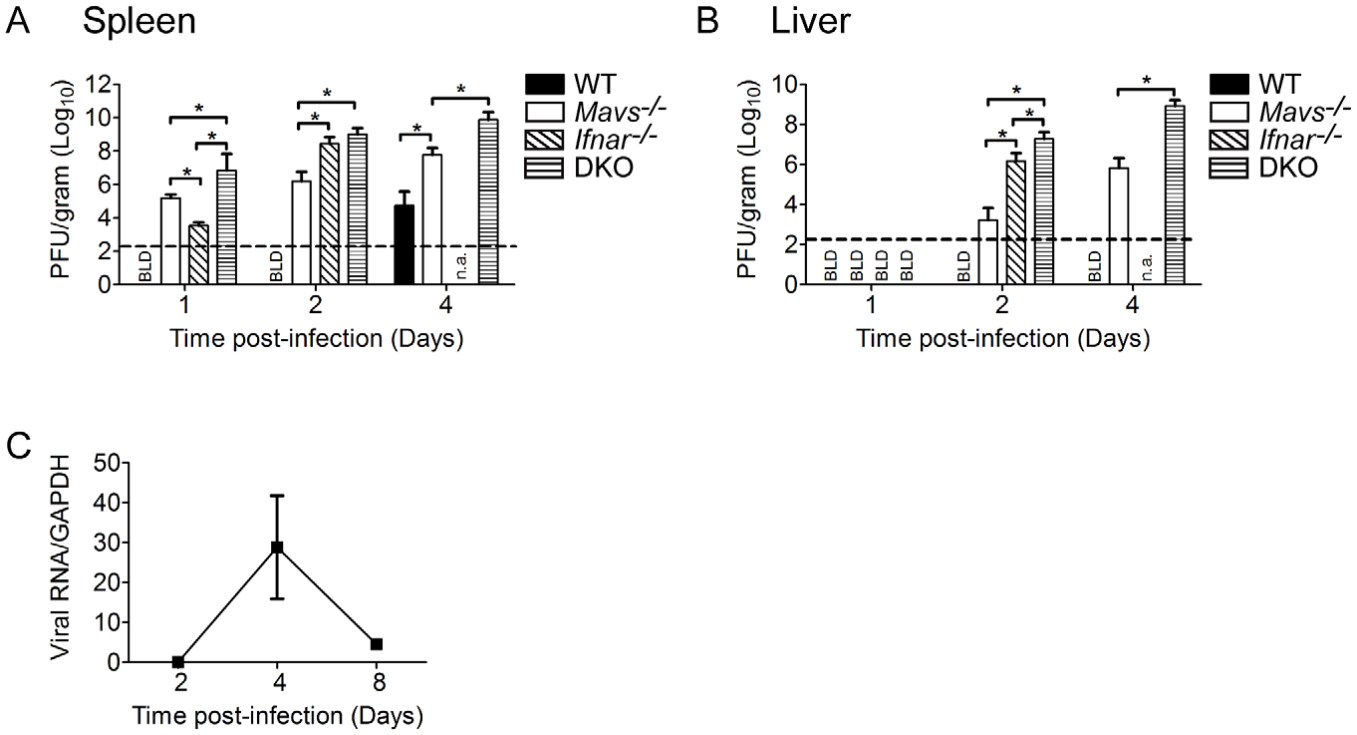
WNV replication in permissive and nonpermissive tissues is controlled by RLR and type I IFN signaling. Viral burden analysis of spleen (permissive) and liver (nonpermissive) tissues from WT, *Mavs^−/−^*, *Ifnar^−/−^*, and *Mavs^−/−^*×*Ifnar^−/−^* (DKO) mice infected subcutaneously in the footpad with 100 PFU of WNV-TX. Viral burden in the (**A**) spleen and (**B**) liver were determined by plaque assay. Data are represented as PFU per gram (n = 4 mice per timepoint). (**C**) Viral burden in WT infected livers determined by qRT-PCR using virus-specific primers and represented as a relative ratio of WNV to GAPDH RNA (n = 3–4) mice per timepoint). Graphs show the mean +/− standard deviation for each measurement. Asterisk denotes *P*<0.05. The horizontal line indicates the lower limit of assay sensitivity. BLD = below limit of detection. n.a. = not applicable.

The innate immune response is critical for controlling WNV replication, both in peripheral organs and the CNS, which ultimately leads to protection from virus challenge. Thus, the lack of virus control can directly lead to reduced survival. To define the roles of the RLR and type I IFN signaling pathways alone and in combination for controlling virus replication, spread, and tropism, we evaluated viral burden in the spleen and liver after WNV challenge. These tissues represent a permissive (spleen) and a nonpermissive (liver) tissue in WT mice and humans. As compared to WT infected mice, spleens from *Mavs* ^−/−^, *Ifnar* ^−/−^, and DKO infected mice overall exhibited higher peak viral burden (Figure 2A). Virus replication was detected as early as day 1 p.i. in the spleens from *Mavs* ^−/−^ and *Ifnar* ^−/−^ infected mice, with spleens from DKO infected mice displaying significantly higher viral loads. Thus, even in the context of the permissive spleen tissue environment, the RLR and type I IFN signaling pathways impart innate immune responses that restrict early virus replication and limit peak viral burden. Similarly, *Mavs* ^−/−^, *Ifnar* ^−/−^, and DKO infected mice exhibited liver infection (Figure 2B), wherein livers from DKO infected mice displayed the highest peak viral burden and infectious virus production as compared to *Mavs* ^−/−^ and *Ifnar* ^−/−^ infected mice. Interestingly, *Mavs* ^−/−^ infected livers showed significantly reduced viral titers as compared to either *Ifnar* ^−/−^ and DKO infected mice, suggesting RLR-independent signaling pathways control WNV replication in the liver. Importantly, no infectious virus was detected in livers from WT infected mice, consistent with other studies that have shown resistance of WNV infection in the livers of WT mice [6], [32]. Through the use of a highly-sensitive qRT-PCR assay, we found that WNV RNA levels in the liver peaked on day 4 p.i. (Figure 2C). While we cannot entirely rule out the possibility of blood contamination attributing to the detection of WNV RNA in the liver, these findings are consistent with a study that showed immunofluorescence staining of WNV antigen in hepatocytes of livers from WT infected mice [33]. These observations suggest that livers from WT mice are exposed to WNV, but infection is either prevented or controlled through restrictions imposed by innate immune programs mediated by RLR and type I IFN-dependent signaling. Moreover, these observations indicate that RLR and type I IFN actions serve to control the extent of WNV infection in permissive/tropic tissue.

### Innate immune signatures of tissue-specific restriction of WNV replication

**Figure 3:**
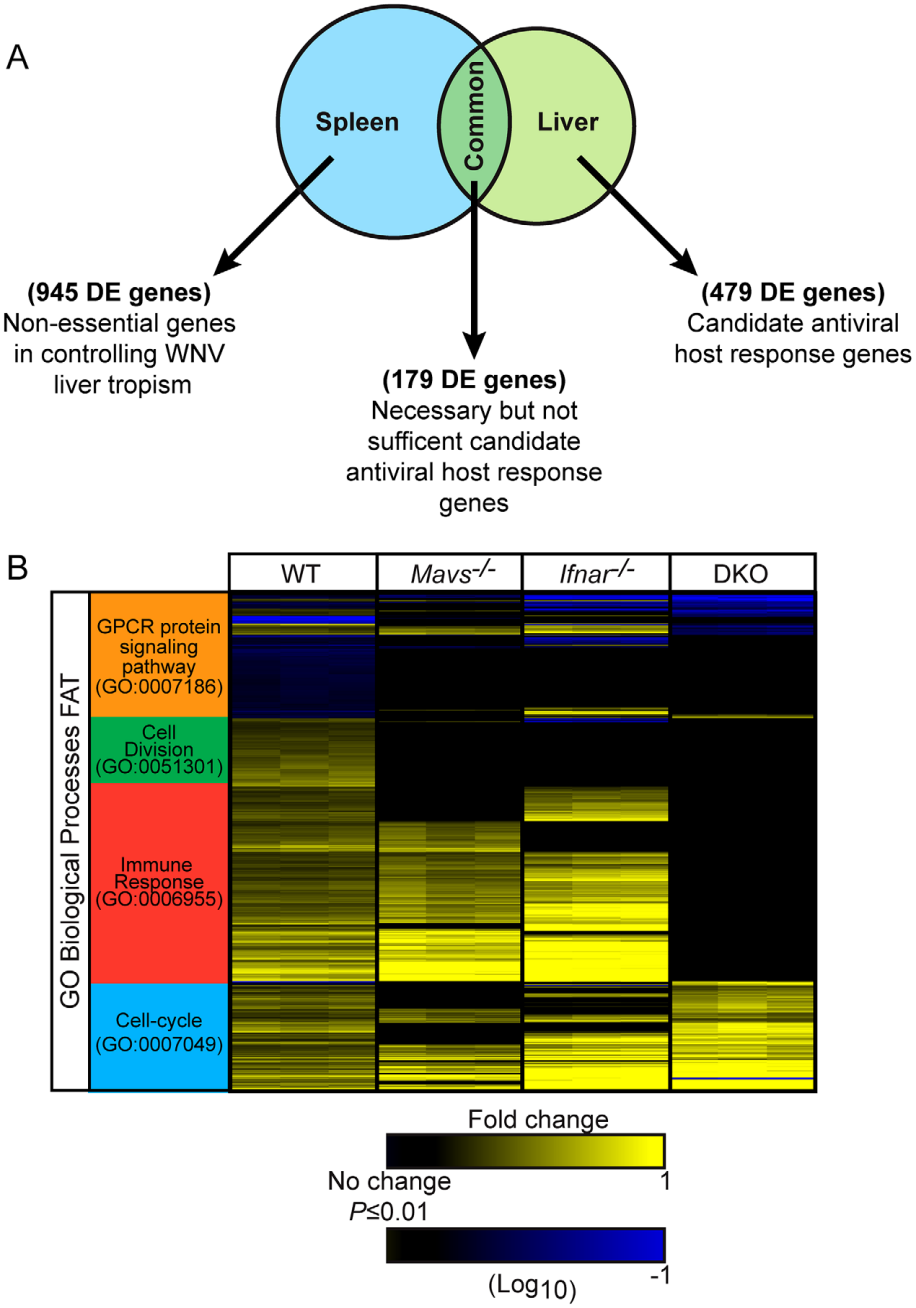
Identification of tissue-specific genes expressed during WNV infection. (**A**) Venn diagram between spleen and liver tissues from WT infected mice. (**B**) Hierarchical clustering of 658 differentially expressed genes between liver from WT and KO infected mouse strains. Differentially expressed genes are shown as log_10_(ratio) of WNV-infected to strain-matched mock-infected mice. Genes whose expression was significantly induced over strain-matched mock-infected mice are depicted in the heat map (1.5 fold; *P*≤0.01). Clustering was performed using hierarchical UPGMA (unweighted average) with Euclidean distance similarity measure in Spotfire DecisionSite. Functional annotation was performed using the DAVID Bioinformatics Resource version 6.7 and gene ontology accession numbers for biological processes FAT are listed. G-protein coupled receptor (GPCR).

To define the gene expression signatures that control permissiveness to WNV infection, we applied mouse whole-genome microarrays to profile global gene expression changes between permissive- (spleen) and nonpermissive- (liver) tissues from WT infected mice on day 4 p.i. Differentially expressed (DE) genes (defined as ≥1.5-fold change in expression with paired Student's t-test *P*≤0.01) were identified by comparing infected spleen and liver to their respective tissues from strain-matched mock-infected controls. Venn analysis defined 3 categories of DE genes whose expression was linked with viral burden in WT infected tissues: 1) Spleen-specific- representing non-restrictive genes in controlling tropism; 2) Common- representing genes that are necessary but not sufficient for controlling tropism; and 3) Liver-specific- representing genes that restrict and control WNV infection tropism (Figure 3A). These analyses yielded 945 spleen-specific DE genes, 179 common DE genes, and 479 liver-specific DE genes. In addition, we performed transcriptional profiling from *Mavs* ^−/−^, *Ifnar^−/−^*, and DKO infected mouse liver to identify genes whose expression is regulated by RLR signaling, type I IFN signaling, or both pathways. We visualized the 658 DE genes from WT infected liver tissues and found that the absence of RLR or type I IFN receptor signaling resulted in reduced gene expression, with the greatest differences in gene expression observed from DKO infected livers (Figure 3B). Hierarchical clustering and functional annotation of liver infected genes revealed that RLR and type I IFN signaling have a significant impact on expression of genes related to G-protein coupled receptor protein signaling pathway (GO:0007186), cell division (GO:0051301) and immune response (GO:0006955) biological processes. Additionally, we identified 359 RLR signaling-dependent DE genes, 281 IFN signaling-dependent DE genes, and 464 RLR and type I IFN signaling-dependent DE genes (Table S1) from WNV infected livers. These gene sets define a signature of RLR and type I IFN signaling target genes that correspond to restricted tissue tropism to WNV.

**Figure 4:**
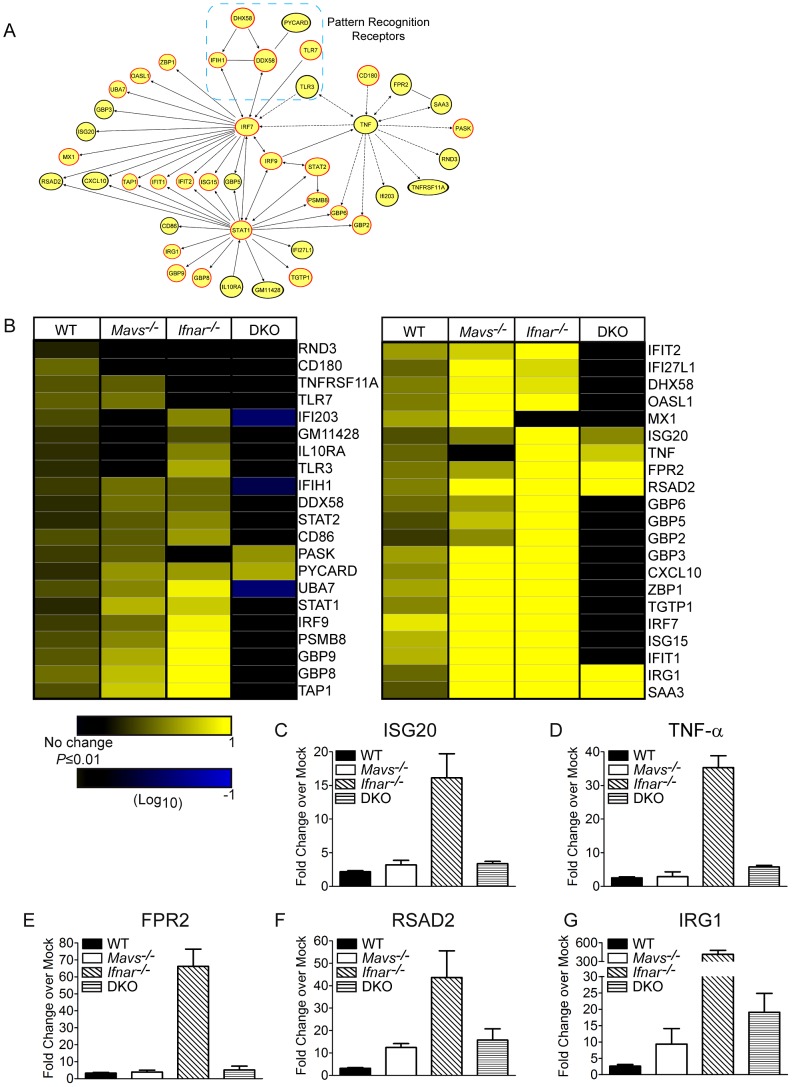
Inflammatory response biological function is highly upregulated in WNV infected liver tissues. (**A**) Network analysis of the top scoring biological function involving inflammatory response from WT infected liver tissue. Genes outlined in red are differentially expressed in both spleen and liver WNV-infected tissues. Genes outlined in black are differentially expressed only in liver WNV-infected tissues. (**B**) Hierarchical clustering of differentially expressed genes derived from the inflammatory response biological function between livers from WT and KO infected mouse strains. Gene expression is shown as log_10_(ratio) of WNV-infected to strain-matched mock-infected mice. Genes whose expression was significantly induced over strain-matched mock-infected mice are depicted in the heat map (1.5 fold; *P*≤0.01). Clustering was performed using hierarchical UPGMA (unweighted average) with Euclidean distance similarity measure in Spotfire DecisionSite. (**C**) ISG20, (**D**) TNF-α, (**E**) FPR2, (**F**) RSAD2, and (**G**) IRG1 expression graphed as fold change over strained matched mock-infected liver tissues.

We next performed computational modeling of gene expression data to define gene networks and their canonical regulatory pathways responsive to WNV infection within WT spleen and liver. DE gene lists were analyzed with Ingenuity Pathway Analysis (IPA). The top five biological categories from this analysis are presented in Table 1. WT infected spleen displayed cell cycle and cancer-related pathways as top-ranking biological processes. This signature is consistent with initiation of cell-mediated innate and adaptive immune responses and their dependence on immune cell proliferation beginning on day 4 p.i. [8], [22]. In contrast, WT infected liver tissues displayed inflammatory response and cell-to-cell signaling and interaction as top ranking biological processes, reflecting the essential nature of the innate immune response in restricting tissue permissiveness to WNV infection. Network analysis of genes comprising the inflammatory response biological function from WT infected livers (Figure 4A) revealed a highly interconnected network consisting of pattern recognition receptors (DDX58 (RIG-I), IFIH1 (MDA5), DHX58 (LGP2), TLR3, and TLR7), IRF-3 and ISRE-target genes with known antiviral effector function against WNV and other RNA viruses (MX1 [34], IFIT1 [15], [16], [35], IFIT2 [36], ISG15 [37], [38], GBP2 [39], and GBP3 [40], innate immune transcription factors (IRF-7, IRF-9, STAT1, and STAT2), proteins related to antigen presentation (CD86, TAP1 and PSMB8), and pro- and anti-inflammatory cytokine and chemokine related genes (TNF-α, CXCL10 and IL-10RA). As a proof of principle, we also independently identified innate immune genes that have previously been shown to control WNV replication and tissue tropism. These include IRF-7 [41], TLR7 [33], [42], TLR3 [43], RSAD2 [19], MDA5 [12], RIG-I [12], IFIT1 [15], IFIT2 [14], [15], ISG20 [17], and OAS [44]. Through network analysis, we defined MDA5, RIG-I, IRF-7, IRF-9, STAT2, STAT1, and TNF-α as regulatory nodes of gene expression (as determined by ranking the total number of edges to a given node) within infected livers. Additionally, we observed distinct tissue-specific gene expression patterns, in which essential innate immune signaling components RIG-I, MDA5, IRF-7, IRF-9, STAT1, and STAT2 were expressed in spleen and liver tissues, whereas TLR3 and TNF-α were only expressed in infected liver tissue. These findings support our hypothesis that distinct tissue-specific innate immune programs restrict tissue tropism to WNV.

**Table 1:** Top biological categories determined by IPA.

Spleen	B-H *P value*
Cell Cycle	6.86E-18 to 1.41E-02
Cancer	4.29E to 16-1.38E-02
Dermatological Diseases and Conditions	3.84E-15 to 1.38E-02
Inflammatory Response	7.47E-15 to 1.47E-02
Antimicrobial Response	6.70E-14 to 1.38E-02

Liver	B-H *P value*
Inflammatory Response	5.25E-38 to 3.66E-03
Cell-To-Cell Signaling and Interaction	6.61E-23 to 3.84E-03
Cellular Growth and Proliferation	9.27E-22 to 3.84E-03
Hematological System Development and Function	9.27E-22 to 3.84E-03
Tissue Morphology	6.49E-19 to 2.45E-03

To identify the innate immune signaling pathways that directly regulate gene expression and restrict liver tropism of WNV, we compared genes within this inflammatory network with expression profiles from *Mavs* ^−/−^, *Ifnar^−/−^*, and DKO infected liver tissues. The absence of MAVS resulted in the loss of expression for a select number of genes, including CD180, IFI203, GM11428, IL10RA, TLR3, and TNF-α (Figure 4B). Surprisingly, in the absence of type I IFN signaling, few genes were found to be reduced or downregulated in expression. Rather, many genes were found to be increased or upregulated in expression as compared to genes from WT or *Mavs* ^−/−^ infected liver tissues. The combined absence of RLR and type I IFN signaling pathways displayed markedly downregulated gene expression as compared to WT or *Mavs* ^−/−^ and *Ifnar* ^−/−^ infected liver tissues. Genes that were expressed to high levels in the *Ifnar* ^−/−^ infected liver tissues were strongly reduced in DKO infected liver tissues (Figure 4C–G), indicating that a major component of the host response to WNV infection triggered in *Ifnar* ^−/−^ infected liver is mediated through RLR signaling. These findings demonstrate that RLR and type I IFN signaling pathways are required to confer complete expression of host response genes for innate immune protection and the restriction of infection within liver tissue.

### Gene network analysis of WNV infected liver tissues

**Figure 5:**
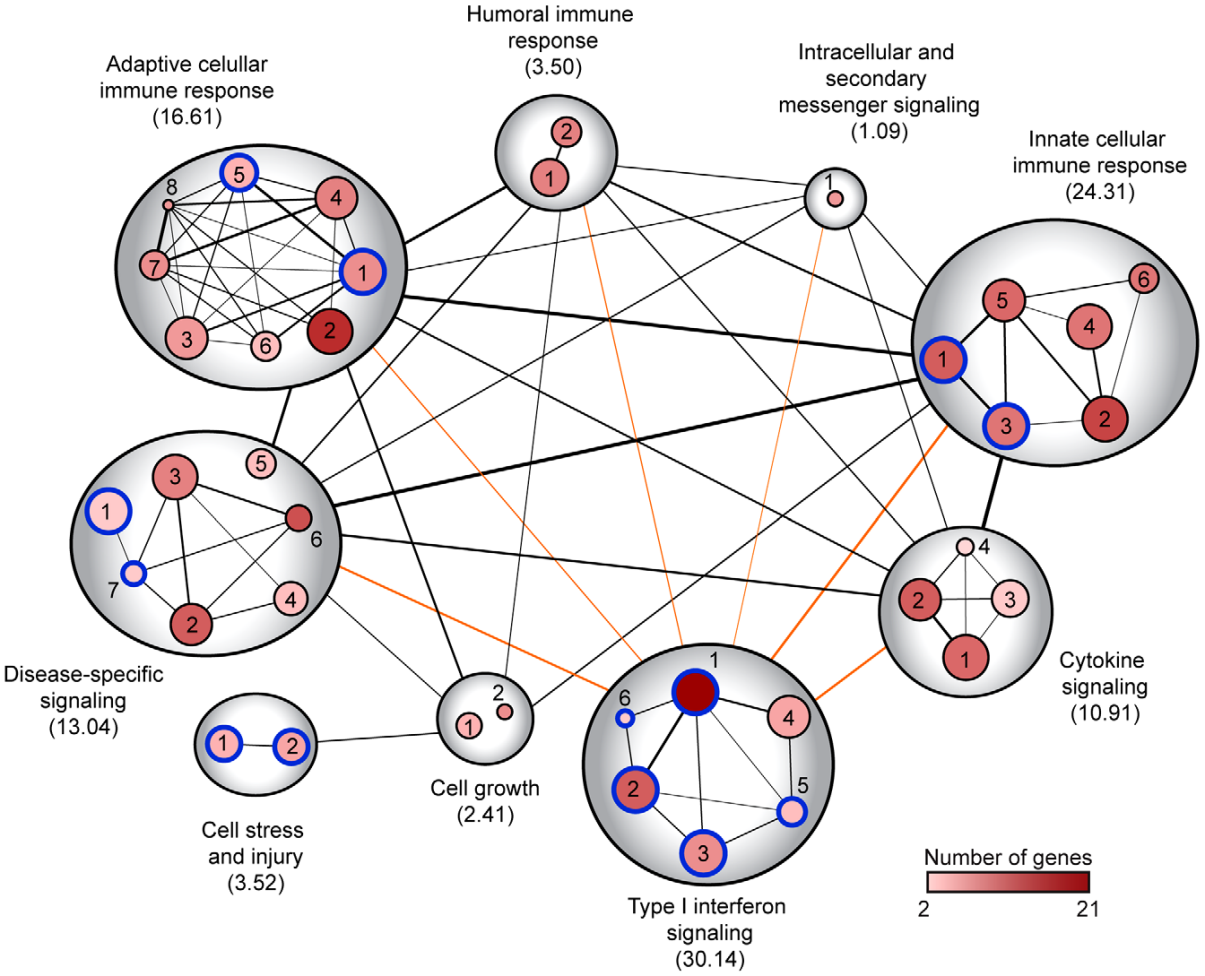
Network enrichment map of wild type WNV infected livers. Enrichment map for gene expression in WT infected livers. Nodes represent top scoring Ingenuity Pathway Analysis curated canonical pathway gene sets significantly enriched over mock-infected mice, and edges between nodes represent overlap between two connected gene sets. Edge thickness is proportional to number of overlapping genes. Node size is proportional to enrichment significance (the larger the node the greater the significance). Node color is proportional to the number of genes comprising the canonical pathway (the darker the node means greater number of genes). Node edges in blue represent common pathways between spleen and liver infected tissues. Nodes edges in black represent liver-specific pathways. Node identity is depicted by the number adjacent to each node and the key is listed in Table S4. Clusters were manually organized, circled, and labeled to highlight the prevalent biological functions among related gene sets. Enrichment scores (−log_10_ B-H *P* value) are depicted under the name of each biological function.

To reveal the relationship between the known cellular pathways enriched in WT infected spleen and liver we applied IPA and computational modeling of curated response pathways. We found that pathways specific to the role of pattern recognition receptors in recognition of bacteria and viruses, activation of IRFs by cytosolic pattern recognition receptors, and interferon signaling were among the top scoring enriched canonical pathways in infected tissue (Tables S2 and S3). To define overlapping gene-sets and network clusters among infected tissues, we visualized the top-ranked IPA pathways from WT infected liver through a network-based Enrichment Map method within Cytoscape [45], in which canonical pathways were organized into networks grouped by major biological function. In this analysis, canonical pathways are represented by a node with edges represent overlapping genes between sets (Figure 5 and Table S4). This visualization method revealed a highly interconnected network of biological processes that are regulated within the WT infected liver. The IFN signaling biological function displayed the highest enrichment score (ES = 30.14), demonstrating that IFN-mediated responses play a strong role in regulating early host responses to WNV infection. Furthermore, the IFN signaling intra-connected canonical pathways (comprised of: 1- role of pattern recognition receptors in recognition of bacteria and viruses, 2- activation of IRF by cytosolic pattern recognition receptors, 3- interferon signaling, 4- toll-like receptor signaling, 5- role of PKR in interferon induction and antiviral response, and 6- role of RIG-I like receptors in antiviral innate immunity) displayed mutually overlapping gene-sets among the major biological functions listed in Figure 5, including gene sets of the innate cellular immune response (ES = 24.31), adaptive cellular immune response (ES = 16.61), disease-specific signaling (ES = 13.04), cytokine signaling (ES = 10.91), humoral immune response (ES = 3.50), and intracellular and secondary messenger signaling (ES = 1.09).

Using enrichment map analysis, we next investigated additional signaling pathways that may regulate liver tropism to WNV by examining the innate cellular immune response regulatory nodes. We identified NK cell signaling (node 2; *P* = 3.39×10^−6^) and NK cell-related signaling pathways, including communication between innate and adaptive immune cells (node 1; *P* = 2.14×10^−6^) and crosstalk between dendritic cells and natural killer cells (node 3; *P* = 2.4×10^−4^; Table S4) as top-scoring canonical pathways. Among these pathways, the NK cell signaling canonical pathway was exclusively enriched in the liver, suggesting that NK cells and related signaling pathways play an important role in the control of WNV tissue tropism and restriction of infection in the liver. Moreover, our data sets indicated that additional signaling pathways, including TREM1 signaling (P = 6.17×10^−7^), Fcγ Receptor-mediated Phagocytosis in Macrophages and Monocytes (P = 1.26×10^−3^), and other top scoring liver-specific canonical pathways might impart further innate immune actions that contribute to restriction of WNV tissue tropism. We also compared gene expression data from KO infected liver tissues to determine what role the RLR and type I IFN signaling pathways play in regulating NK cell signaling. The NK cell signaling pathway was enriched in livers from *Mavs* ^−/−^ and *Ifnar* ^−/−^ infected mice, but not from DKO infected mice, suggesting an important connection between the RLR and type I IFN signaling axis and NK cell regulation (Table 2).

**Table 2:** Linkages with the RLR and type I IFN signaling axis.

Top IPA Canonical Pathways (MAVS dependent DE genes)	B-H *P value*	# of Molecules	Ratio
TREM1 Signaling	2.14E-05	7	0.12
Diff. Reg. of Cytokine Production in Intestinal Epithelial cells by IL-17A and IL-17F	1.29E-03	4	0.18
Role of Pattern Recognition Receptors in Recognition of Bacteria and Viruses	1.29E-03	6	0.07
Altered T Cell and B Cell Signaling in Rheumatoid Arthritis	1.66E-03	6	0.07
Diff. Reg. of Cytokine Production in Macs and Th Cells by IL-17A and IL-17F	9.77E-03	3	0.17

Top IPA Canonical Pathways (type I IFN dependent DE genes)	B-H *p value*	# of Molecules	Ratio
Role of Pattern Recognition Receptors in Recognition of Bacteria and Viruses	1.82E-02	5	0.06
Glycosphingolipid Biosynthesis – Globoseries	2.29E-02	3	0.14

Top IPA Canonical Pathways (MAVS and type I IFN dependent genes)	B-H *p value*	# of Molecules	Ratio
Role of Pattern Recognition Receptors in Recognition of Bacteria and Viruses	2.63E-06	10	0.12
TREM1 Signaling	2.09E-04	7	0.12
Leukocyte Extravasation Signaling	5.50E-04	11	0.06
Natural Killer Cell Signaling	9.33E-04	8	0.08
iCOS-iCOSL Signaling in T Helper Cells	9.33E-04	8	0.07

### NK cell signaling is regulated by the RLR and type I IFN signaling axis

**Figure 6:**
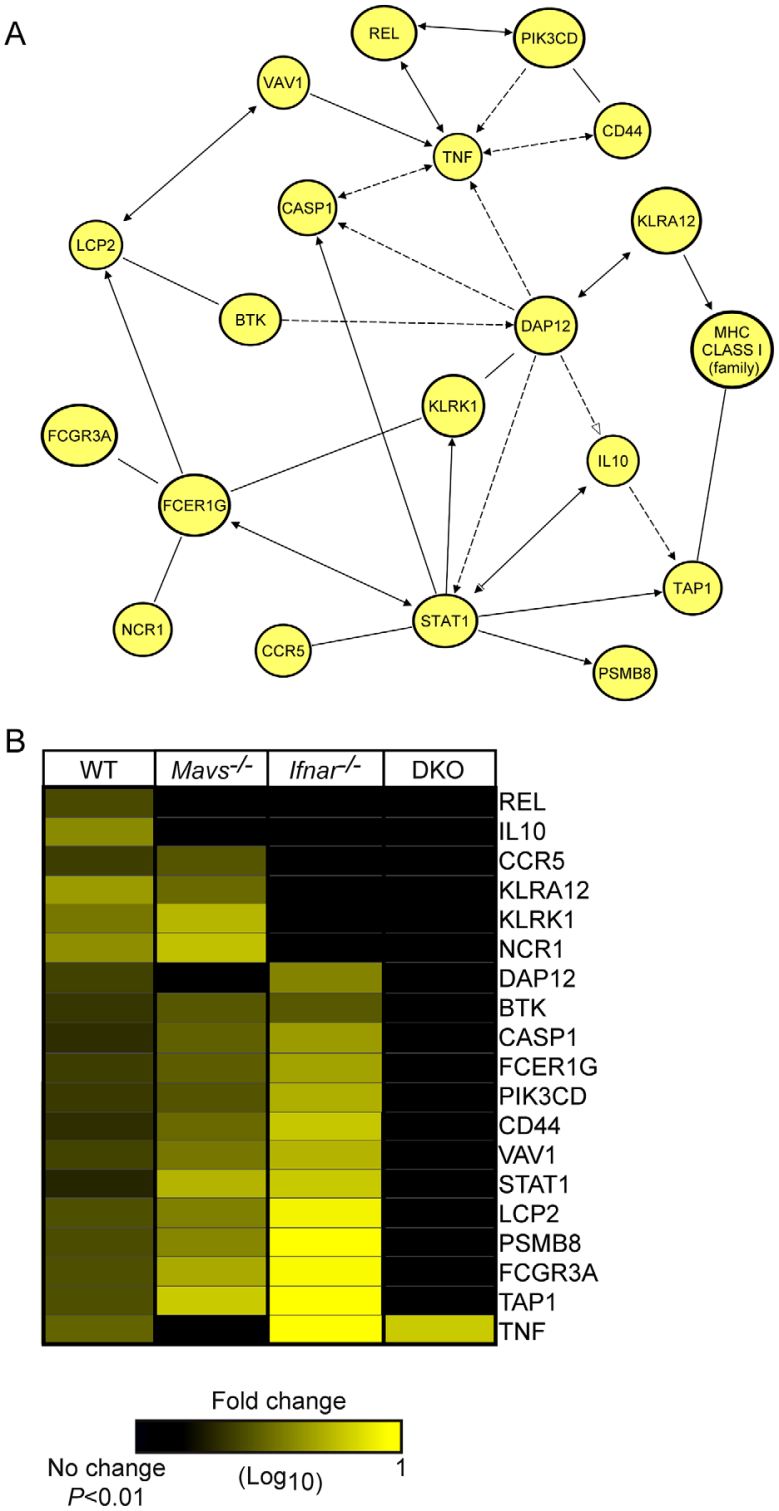
NK cell signaling controls WNV liver replication. (**A**) Network analysis of genes induced in WT infected liver tissues comprising the NK cell signaling canonical signaling pathway. (**B**) Hierarchical clustering of differentially expressed genes between WT and KO infected mouse strains. Gene expression is shown as log_10_(ratio) of WNV-infected to strain-matched mock-infected mice. Genes whose expression was significantly induced over strain-matched mock-infected mice are depicted in the heat map (1.5 fold; *P*≤0.01). Clustering was performed using hierarchical UPGMA (unweighted average) with Euclidean distance similarity measure.

NK cells are important components of the innate immune response that regulate viral infection by killing infected cells and regulating inflammatory cytokine milieu [46]. However, the role of NK cells in regulating tissue tropism to WNV infection or in controlling WNV replication has not been defined. In the context of WT infected livers (Figure 6), analysis of the NK cell signaling network revealed upregulated expression of molecules that mediate inflammatory cytokine production (REL, STAT1, DAP12, and CD300), detection of “missing self” (MHC class I complex, TAP1, and PSMB8), NK cell activation (CD44, VAV1, BTK, and LCP2), activation of antibody-dependent cell cytotoxicity (FCER1G, FCGR3A), secretion of cytokines (TNF-α, and IL10) and regulatory receptors (KLRK1, KLRA12, and NCR1). As expected, *Mavs^−/−^* or *Ifnar^−/−^* infected livers displayed minor alterations in gene expression within the NK cell signaling network as compared to WT infected livers. Strikingly, DKO infected livers displayed significantly reduced expression for nearly every gene within the NK cell signaling pathway. These findings further suggest that NK cell responses within the liver could be directly regulated by the RLR and type I IFN signaling axis.

### WNV infection triggers expansion of NK cells in the liver

**Figure 7:**
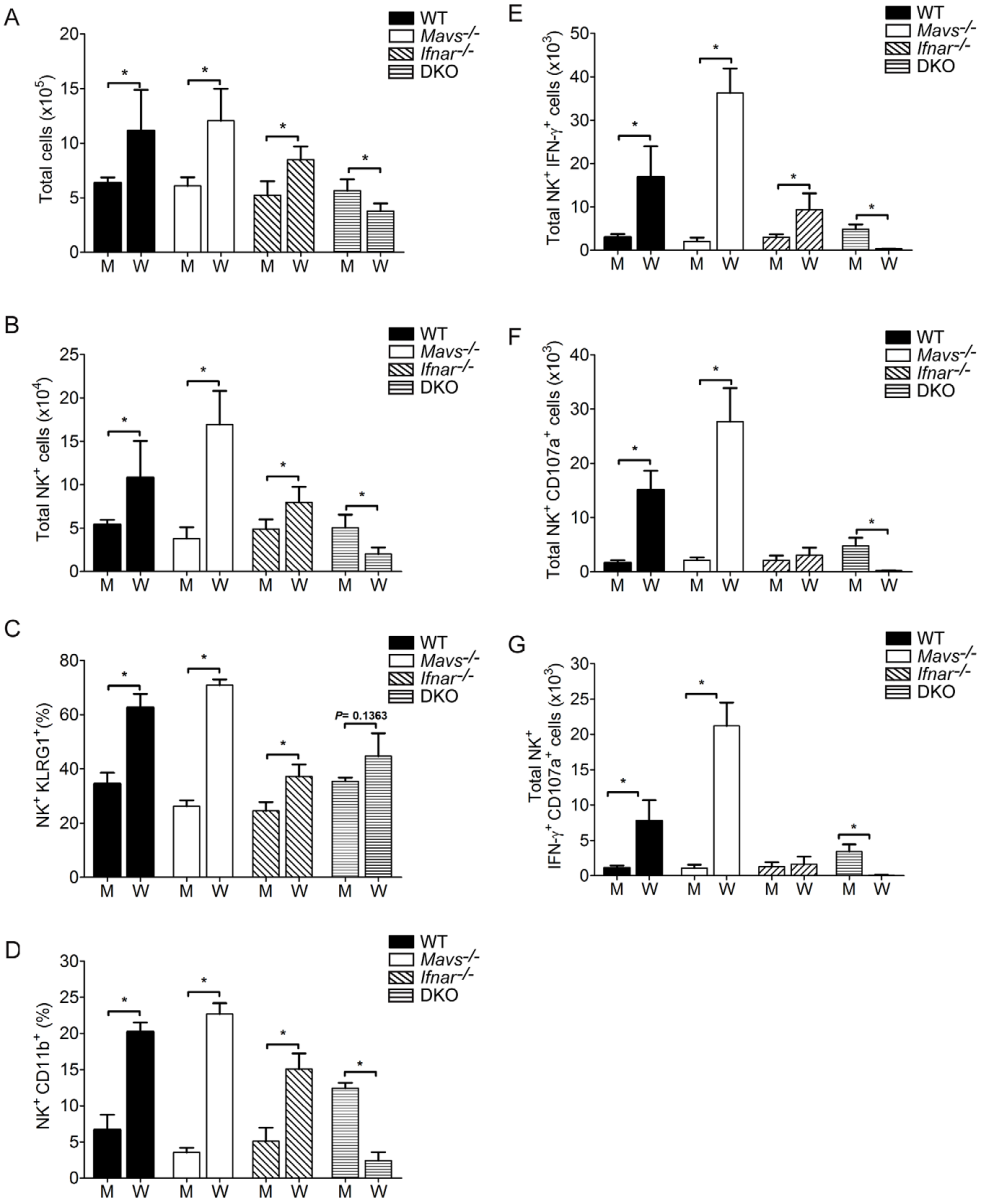
NK cells expand and are functionally active from livers from WT infected mice. WT and DKO mice were mock-infected or infected with WNV-TX. Livers were harvested on day 4 p.i. and immune cells were isolated and characterized by flow cytometry. (**A**) Total cells (**B**) Total number of NK cells (CD3ε^−^NK1.1^+^), (**C**) Percent KLRG1^+^ NK cells, (**D**) Percent CD11b^+^ NK cells, (**E**) Total number of IFN-γ^+^ NK cells, (**F**) Total number of CD107a^+^ NK cells, (**G**) Total number of IFN-γ^+^CD107a^+^ NK cells. Graphs show the mean +/− standard deviation (n = 4). Asterisks denote *P*<0.05. The data are representative of two or more independent experiments.

We next performed biological validation studies to determine the extent of NK cell expansion and activation in livers from WNV-infected mice. On day 4 p.i., livers from WT infected mice displayed significantly increased immune cell infiltrates and NK cells (CD3ε^−^NK1.1^+^) as compared to mock-infected WT mice (Figure 7A–B), indicating that WNV infection triggers a liver NK cell response. Livers from *Mavs^−/−^* and *Ifnar^−/−^* infected mice also showed significant increases in NK cell numbers as compared to their respective mock infected mice. Remarkably, DKO infected mice displayed dramatically reduced numbers of NK cells in the liver as compared to mock infected mice, demonstrating that RLR and type I IFN signaling is essential in regulating liver NK cell recruitment and/or expansion during WNV infection. Phenotypic analysis revealed enhanced proliferation (KLRG1^+^ expression; Figure 7C) and maturation (CD11b^+^ expression; Figure 7D) on liver NK cells recovered from WT infected mice as compared to gene-knockout infected mice. Furthermore, liver NK cell subsets from WT infected mice displayed enhanced IFN-γ and/or CD107a (a sensitive marker for NK cell functional activity, including cytokine secretion and NK cell-mediated lysis [47]) positive phenotype as compared to NK cells from *Ifnar^−/−^* and DKO, but not *Mavs^−/−^*, infected mice (Figures 7E–G). These findings corroborate our computational and pathway analyses that found specific enrichment for the NK cell signaling pathway within WT infected livers as compared to DKO infected livers. Overall, these findings provide biological validation that the RLR and type I IFN signaling axis is important in regulating NK cell recruitment, proliferation, and effector functions within the livers of WNV infected mice.

### RLR and type I IFN signaling regulate NK cell effector function

**Figure 8:**
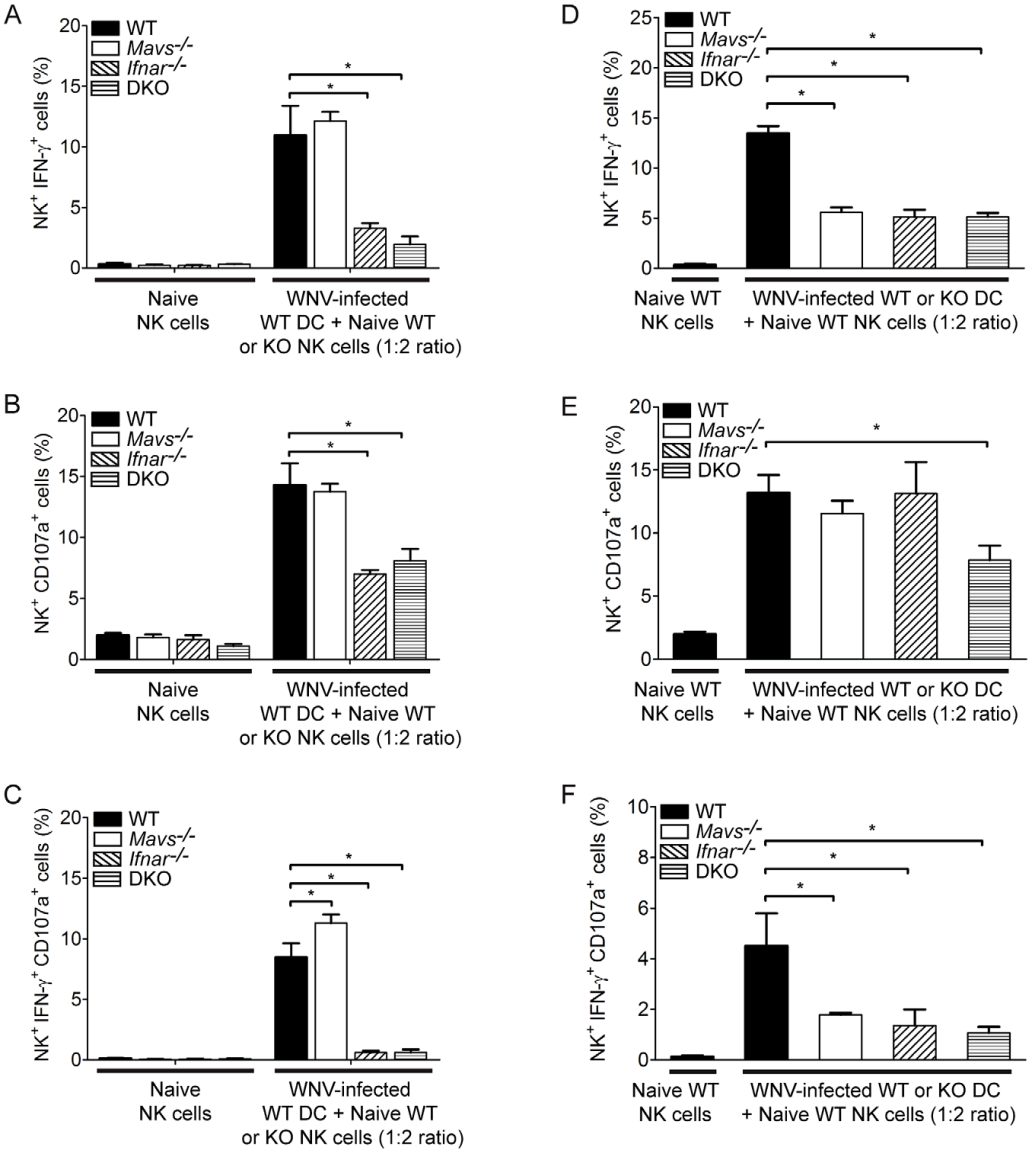
Type I IFN functions in a cell-intrinsic manner to regulate NK cell effector functions. (**A–C**) Bone-marrow derived dendritic cells from WT mice were infected at an MOI of 5.0 with WNV-TX and cultured together with purified NK cells (CD3ε^−^NK1.1^+^) from either WT, *Mavs^−/−^*, *Ifnar^−/−^*, and DKO mice. At 18 hours post-coculture, cells were collected, stained, and analyzed by flow cytometry. As a control, a subset of NK cells were analyzed prior to coculture with BM-DCs (naïve). (**A**) Percent IFN-γ^+^, (**B**) Percent CD107a^+^, (**C**) Percent IFN-γ^+^CD107a^+^ NK cells are shown. Bone-marrow derived dendritic cells from WT, *Mavs^−/−^*, *Ifnar^−/−^*, and DKO mice were infected at an MOI of 5.0 with WNV-TX and cultured together with purified natural killer cells from WT mice and evaluated by flow cytometry. (**D**) Percent IFN-γ^+^, (**E**) Percent CD107a^+^, (**F**) Percent IFN-γ^+^CD107a^+^ NK cells are shown. Graphs show the mean +/− standard deviation (n = 3). Asterisks denote *P*<0.05. The data are representative of two or more independent experiments.

Crosstalk between dendritic cells and natural killer cells is essential for NK cell activation during viral infection [48]. Our computational modeling revealed specific enrichment for NK cell processes, including the canonical pathway for DC/NK cell crosstalk (see Figures 5 and 6A). As DCs are a target cell for WNV infection *in* vivo, we sought to establish whether the RIG-I like receptor or IFN signaling pathways function in a cell-intrinsic or cell-extrinsic manner to regulate NK cell effector functions through DC/NK cell crosstalk during infection. We evaluated this using a coculture assay wherein NK cells are cultured together with bone-marrow derived DCs and assayed for NK cell effector function [48]. Culture of WT WNV-infected BM-DCs with syngeneic purified WT NK cells (1∶2 ratio) triggered NK cell effector function, as determined by NK cell expression of intracellular IFN-γ (Figure 8A), CD107a (Figure 8B) or induction of both IFN-γ and CD107a (Figure 8C) as compared to NK co-culture with naïve control DCs. Cocultured *Mavs^−/−^* NK cells with WT WNV-infected DCs exhibited similar enhancement of IFN-γ and CD107a expression as compared to WT NK cells, demonstrating that MAVS does not act in a cell-intrinsic manner to regulate NK cell effector function during WNV infection. In contrast, *Ifnar^−/−^* and DKO NK cells cocultured with WT WNV-infected DCs exhibited an attenuated effector phenotype, demonstrating that IFN functions in a cell-intrinsic manner to regulate NK cell effector function during WNV infection. In a reciprocal set of cocultures, *Mavs^−/−^*, and *Ifnar^−/−^* WNV-infected DCs exhibited reduced ability to trigger IFN-γ secretion, but not degranulation, in NK cells (Figures 8D–F). DKO WNV-infected DCs exhibited a significant reduction in the ability to activate NK cell effector functions, including IFN-γ secretion and CD107a expression (Figures 8D–F). Combined with our *in vivo* NK cell analysis, we conclude that both the RLR and type I IFN signaling pathways act in a NK cell-extrinsic manner, whereas IFN acts in a NK cell-intrinsic manner, to activate NK cell effector functions during viral infection. Overall, these observations reveal a complex innate immune signaling network, regulated by the RLR and type I IFN signaling pathways, that drives tissue-specific innate immune programs to control tissue tropism to WNV infection. Furthermore, these studies provide novel insight into the regulation of hepatic tropism to WNV infection through the expression of antiviral effector genes and innate immune cellular processes.

## Discussion

**Figure 9:**
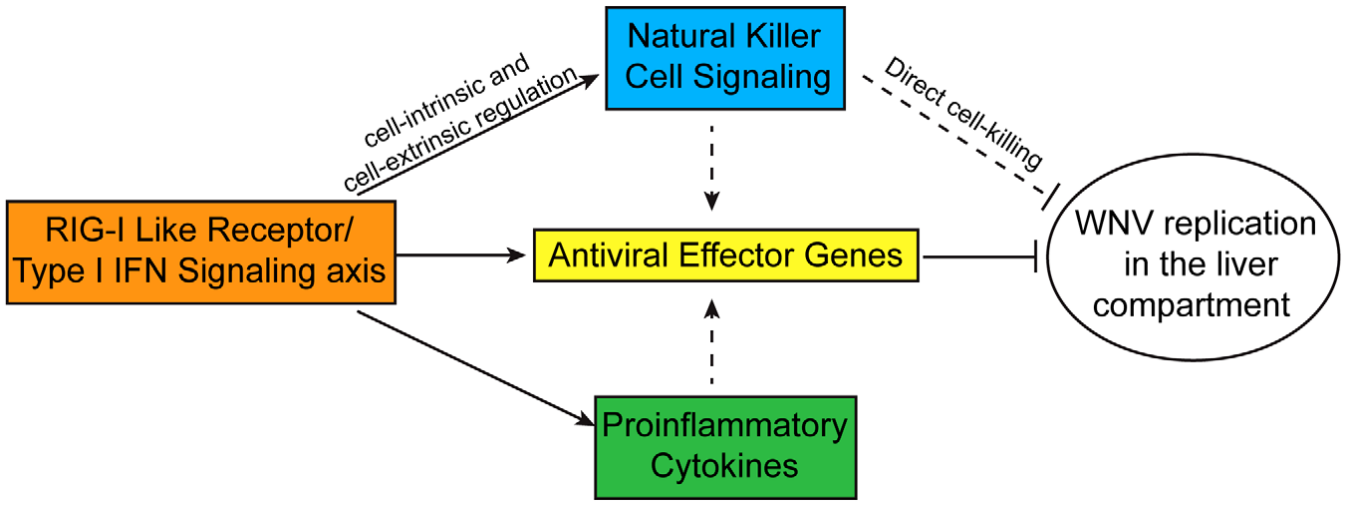
A model illustrating the host signaling pathways that regulate liver tropism to WNV. Upon WNV infection, the RLRs trigger expression of type I IFN, leading antiviral effector gene expression, pro-inflammatory cytokine induction, and activation of NK cells and related signaling pathways. Antiviral gene expression can further be amplified by natural killer cells and induction of pro-inflammatory cytokines, such as TNF-α and IFN-γ. A combination of antiviral gene expression and natural killer cells, through direct cell lysis, participate in regulating WNV replication in the liver.

Innate immunity is our first line of defense against viral pathogens and serves to protect us from infection on a daily basis. The proper coordination of innate immune signaling pathway function and efficient expression of antiviral effector genes are key to governing the outcome of virus infection and immunity. In this study, we demonstrate that the RLR and type I IFN signaling axis plays an essential role in protection against WNV infection and functions in a tissue-specific manner to control virus replication and restrict tissue tropism. We demonstrate that WNV can indeed infect liver tissue but this infection is rapidly cleared in WT infected mice but not RLR-deficient and/or IFN signaling-deficient mice. In order to define the genomic signature and components of a protective innate immune response that restricts WNV infection, we applied an integrated systems biology approach to uncover novel connections between gene networks and pathways. Functional genomics analysis and pathway modeling revealed that inflammatory response programs were highly enriched in WT WNV-infected liver compared to the permissive spleen. As a proof of principle, we identified genes encoding known innate antiviral effectors against WNV [14]–[20], including pattern recognition receptor and interferon signaling pathways [8], [13]. In addition, we revealed that the NK cell signaling network is an important feature of innate immune restriction of WNV infection. Through pathway modeling and biological studies, the NK cell responses were found to be directly regulated by the RLR and type I IFN signaling axis. Our observations support a model in which the gene networks within the RLR and type I IFN signaling axis, including IRFs, STATs, and ISG effectors, impart restriction of virus replication while linkage with innate immune cellular process, such as with NK cell signaling, facilitate the innate immune response. Ultimately, this complex signaling network controls WNV infection and prevents virus spread within the liver (Figure 9). While NK cells from livers of WNV infected WT mice were fully mature, proliferating, and functional, NK cells from DKO infected liver tissues were dysregulated, exhibiting reduced expression of maturation markers, reduced proliferation, and attenuated effector functions. In contrast to infected livers, gene network analysis revealed that the permissive environment of the spleen for WNV infection displayed high enrichment for IFN and related signaling pathways. Interestingly, we observed significant enrichment of the canonical pathway involved in crosstalk between DCs and NK cells in the spleen and this enrichment was consistent with an increase in splenic NK cells in WT infected mice (data not shown). However, we did not observe spleen-specific enrichment for NK cell interactions or related signaling pathways, suggesting that NK cell-dependent signaling is essential in hepatic but not splenic control of WNV infection. Overall, this study identified host factors and their biological processes that control liver tropism, and defined key linkages between the RLR and type I IFN signaling axis and signaling of NK cell effector function.

An unexpected and exciting finding from this study was that DKO mice lived longer than *Ifnar^−/−^* mice. This increase in average WNV infection survival time correlated with stark differences in gene expression profiles in permissive and nonpermissive tissues from these knockout mice, wherein *Ifnar^−/−^* infected tissues displayed a robust inflammatory response as compared to DKO infected tissues that showed a dramatic loss in inflammation. This finding suggests that *Ifnar^−/−^* infected mice are likely succumbing to infection from a combination of inflammation-mediated and virus-mediated pathology. In contrast, DKO infected mice are likely succumbing from virus-mediated pathology as uncontrolled virus replication linked with a stark reduction in overall inflammatory response. An intriguing question is why do DKO infected tissues show such reduced inflammatory responses as compared to either *Mavs^−/−^* or *Ifnar^−/−^* infected tissues, despite the presence of RLR-independent signaling pathways, including TLR, Nod-like receptor, and C-type lectin receptors? Despite the increased viral burden within *Mavs^−/−^* infected livers, we observed minor changes in inflammation, as defined by expression of inflammatory and antiviral effector genes (Figure 4), as compared to WT infected livers. While this response is unable to effectively control WNV replication within the liver, these findings do suggest that RLR-independent signaling pathways participate in triggering a host inflammatory response to WNV infection. Indeed, analysis of *Ifnar^−/−^* infected livers suggest that RLR-dependent and -independent signaling pathways are responsible for inducing an inflammatory response, despite the lack of type I IFN signaling. Remarkably, livers from DKO mice showed abrogated inflammation, revealing novel crosstalk between RLR-dependent, RLR-independent, and type I IFN signaling pathways that impart an antiviral inflammatory response.

Previous studies of conventional infection-based analysis of knockout mice [4] and gene over-expression or silencing studies in vitro [17], [18] identified specific restriction factors that play an important role in controlling WNV infection and pathogenesis. These genes include those encoding pattern recognition receptors (RIG-I, MDA5, Toll-like receptor (TLR) 3, TLR7, PKR), antiviral effector proteins (ISG20, IFIT1, IFIT2, and RSAD2), transcription factors (IRF-7, IRF-9, STAT1, and STAT2), and pro-inflammatory cytokines (type I IFNs, IFN-γ, TNF-α, CXCL10, and IL10) that inhibit various steps of WNV infection [3], [4], [12], [14], [15], [17], [19], [49]. Our unbiased systems approach now confirms the tissue-specific role for these factors, while importantly revealing novel candidates of virus restriction, including IFI203, IFI27, GBP2, GBP3, GBP5, GBP6, GBP8, GBP9, FPR2, and IRG1. When categorizing putative antiviral effector genes based on tissue-specific expression patterns (permissive versus nonpermissive tissue), we found that expression of the aforementioned restriction factors are linked directly to RLR signaling or type I IFN signaling pathways [17], [18]. Moreover, as our observations reveal a novel linkage of NK cell signaling with the RLR and type I IFN signaling axis, we conclude that efficient innate immune response requires coordinated signaling through multiple innate immune pathways that extend well beyond any single host restriction factor.

NK cells are an important component of the innate immune response and are essential in bridging the innate and adaptive immune responses [50]. These cells have been shown to be crucial for immune defense against viral pathogens, including mouse cytomegalovirus [46], lymphocytic choriomeningitis virus [51], WNV [52], Herpes viruses [53], Hepatitis C virus [54], and Influenza virus [55]. We note that a direct role for NK cells in controlling WNV infection *in vivo* has yet to be demonstrated, though early control of virus replication and tissue permissiveness has been linked to perforin and IFN-γ, two host factors that are actively secreted by NK cells and other innate immune cells [56], [57]. We attempted to deplete NK cells using an antibody-based approach, however, we found that NK cells can only be fully depleted from spleens, but not liver at early times post-WNV infection (data not shown). These results confirm our informatics analysis that NK cells or NK cell signaling are not required for controlling WNV replication in the spleen. At this time, we are unable to conclusively demonstrate an essential role for NK cells in controlling WNV liver tropism *in vivo*. Nonetheless, West Nile and Dengue virus E protein directly bind to the natural cytotoxicity receptor NKp44, expressed on human NK cells, leading to IFN-γ secretion and cytolytic activity [58]. Our pathway modeling suggested that WNV infection triggers liver-specific NK cell activation that is directly regulated by the RLR and type I IFN signaling axis. In fact, our biological validation studies found that infection of WT mice triggered expansion and activation of NK cells within the liver but this response was markedly reduced in DKO infected mice. Furthermore, *in vitro* coculture studies demonstrated that IFN signaling, rather than RLR signaling, function in an NK cell-intrinsic manner to regulate effector functions. It is remains plausible that the RLR signaling pathway supports NK cell activation and maturation during WNV infection since NK cell responses from livers of DKO mice were strongly reduced as compared to NK cells from *Ifnar^−/−^* infected mice. With respect to IFN, our findings are consistent with previous studies that have shown that IFN functions in a NK cell-intrinsic manner to regulate NK cell activation, proliferation, and maturation [29]. These findings are consistent with other independent studies which found that MAVS functions in a NK cell-extrinsic manner, specifically in dendritic cells and diverse accessory cells, to regulate NK cell activity. This suggests that type I IFN production by DCs imparts signaling crosstalk to NK cells that drives their activation [59]–[61]. Our findings expose a novel linkage between the RLR and type I IFN signaling axis with the regulation of NK cell expansion and effector activity during WNV infection.

The use of our integrated systems biology approach has uncovered tissue-specific host restriction factors that control permissiveness to WNV infection. These studies have uncovered novel linkages between the RLR and type I IFN signaling axis while identifying key innate immune regulatory nodes that can be considered as targets for pharmacological interventions. Our findings have significant implication for the design of strategies to target host restriction factors and pathways for antiviral therapies against flavivirus infections.

## Materials and Methods

### Ethics statement

This study was carried out in accordance with the recommendations in the Guide for the Care and Use of Laboratory Animals of the National Institutes of Health. Animal experiments were approved and performed in accordance to the Institutional Animal Care and Use Committee at the University of Washington (Protocol Number: 4158-01).

### Cells and viruses

BHK-21 cells were cultured in Dulbecco's modified Eagle medium (DMEM) supplemented with 10% fetal bovine serum (FBS), HEPES, L-glutamine, sodium pyruvate, antibiotic-antimycotic solution, and nonessential amino acids. WNV isolate TX 2002-HC (WNV-TX) was described previously [62] and tittered by a standard plaque assay on BHK-21 cells. Working stocks of passaged WNV-TX [8] or WNV-TX infectious cloned virus [63] were generated in BHK-21 cells as previously described and used in both *in vitro* and *in vivo* experiments.

### Mouse experiments

*Sti* ^−/−^ mice (C57BL/6 background; referred to in text as *Mavs^−/−^*) were generated in the Gale laboratory [30]. *Ifnar1^−/−^* mice were generously provided by Murali-Krishna Kaja (C57BL/6 background; referred to in the text as *Ifnar^−/−^*). C57BL/6 wild type inbred mice were commercially obtained (Jackson Laboratories, Bar Harbor, ME). All mice were genotyped and bred in specific pathogen-free conditions in the animal facility at the University of Washington. The methods for mice use and care were performed in accordance with the University of Washington Institutional Animal Care and Use Committee guidelines. Age-matched six to twelve week old mice were inoculated subcutaneously in the left rear footpad with 100 PFU of WNV-TX in a 10 µl inoculum diluted in Hanks balanced salt solution (HBSS) supplemented with 1% heat-inactivated FBS. Mock infected mice were inoculated in a similar manner with diluents alone. Mice were monitored daily for morbidity and mortality.

### Viral tissue burden and quantification

For *in vivo* studies, infected mice were euthanized, bled, and perfused with 20 ml of phosphate-buffered saline (PBS). Spleen, kidney, and livers were removed, weighed, and homogenized in 500 ul of PBS containing 1% heat-inactivated FBS using a Precellys 24 at 1500 RPM for 20 seconds (Bertin Technologies, France). Sample homogenates were tittered by plaque assay on BHK-21 cells. For qRT-PCR analysis of viral load, RNA was extracted from tissues as described below and WNV RNA copy number was measured by RT-quantitative PCR (RT-qPCR) as previously described [8].

### RNA preparation and oligonucleotide microarray processing

Expression oligonucleotide arrays were performed on RNA isolated from spleen and liver tissues from strain and time-matched mock infected mice (n = 2) and WNV-TX infected WT (n = 3; day 4 p.i.), *Mavs^−/−^*(n = 3; day 4 p.i.), *Ifnar^−/−^* (n = 3; day 2 p.i.), and *Mavs^−/−^*×*Ifnar^−/−^* (n = 3; day 4 p.i.) mice. RNA was extracted from mock-infected and WNV-TX infected tissues using a combination of TRIzol (Life Technologies) with RNAlater solution (Ambion) according to the manufacturer's instructions. RNA was further purified using RNeasy columns (Qiagen). RNA samples were spectroscopically verified for purity, and the quality of the intact RNA was assessed using an Agilent 2100 Bioanalyzer. cRNA probes were generated from each sample by the use of an Agilent one-color Low Input Quick Amp labeling kit (Agilent Technologies). Individual cRNA samples were hybridized to Agilent mouse whole-genome oligonucleotide 4-by-44 microarrays (G4122F; approximately 39,000 unique mouse genes) according to the manufacturer's instructions. Slides were scanned with an Agilent DNA microarray scanner, and the resulting images were analyzed using Agilent Feature Extractor version 8.1.1.1. This software was used to perform image analysis, including significance of signal and spatial detrending and to apply a universal error model. For these hybridizations, the most conservative error model was applied.

### Microarray analysis and bioinformatics

Raw data were then loaded into a custom-designed laboratory information management system (LIMS). Data were warehoused in a Labkey system (Labkey, Inc., Seattle, WA) and analyzed using GeneData Analyst 2.2.1 software (GeneData Solutions In Silico, San Francisco, CA), and Spotfire DecisionSite for Functional Genomics 9.1 software (Tibco Spotfire, Somerville, MA). Raw microarray data have been deposited in NCBI's Gene Expression Omnibus under GEO Series accession number GSE39259 and are also accessible through the Katze Lab website (www.viromics.washington.edu) in accordance with proposed Minimum Information About a Microarray Experiment (MIAME) standards. A Student's t-test (*P*≤0.01) was performed to determine the genes that had significantly different expression levels with infection compared to levels in mock infections for each of the four mouse strains (1.5 fold change). Functional analysis of statistically significant gene expression changes was performed with the DAVID Bioinformatics Resources [64], [65] and Ingenuity Pathways Analysis (IPA; Ingenuity Systems). These softwares analyze expression data in the context of known biological response and regulatory networks as well as other higher-order response pathways. Ingenuity functional analysis identified biological functions and/or diseases that were most significant. For all analyses, a Benjamini-Hochberg test correction was applied to the IPA-generated *P* value to determine the probability that each biological function assigned to that data set was due to chance alone. In the functional networks, genes are represented as nodes, and the biological relationship between two nodes is represented as an edge (line). All edges are supported by at least one published reference or from canonical information stored in the Ingenuity Pathways Knowledge Base.

### Liver immune cell infiltrate analysis

Age-matched six to twelve week old mice were inoculated subcutaneously in the left rear footpad with either diluents alone or 100 PFU of WNV-TX and mice were euthanized and perfused with 20 ml of cold phosphate-buffered saline (PBS). Whole livers were dissected and placed in a 2 ml of cold complete RPMI media (cRPMI; 10% fetal bovine serum, L-glutamine, Non-essential amino acids, sodium pyruvate, and antibiotics/antimycotic). For isolation of liver immune cells, whole livers were mechanically disrupted with glass slides in cRPMI media, triturated, and digested with 0.25 mg/ml Collagenase B (Roche) and 1 U/ml type I DNase in cRPMI media at 37°C for 45 min. Immune cells were isolated after gradient centrifugation from a 44/56% Percoll (Sigma Aldrich) interface, performed red blood cell lysis, washed twice with FACS (PBS with 1% heat-inactivated fetal bovine serum) staining buffer and counted. Immune cells were stained with directly-conjugated antibodies specific to APC-CD3ε (eBiosciences), PE-NK1.1 (eBiosciences), PE-Cy7 CD11b (Biolegend), FITC-KLRG1 (Biolegend), and APC-Cy7-CD107a (Biolegend). Intracellular IFN-γ staining was performed as previously described [8]. Briefly, lymphocytes were washed and stained with cell surface markers followed by permeabilization-fixation using the Cytofix-Cytoperm Kit (BD-PharMingen) and stained with either a Pacific Blue- (ebiosciences) or PE-Cy7-conjugated IFN-γ antibody (Biolegend) at 4°C for 30 min, washed and analyzed by flow cytometry. Flow cytometry was performed on a BD LSRII machine using BD FACSDiva software. Cell analysis was performed on FlowJo (v.9.5.2) software.

### DC/NK cell coculture

NK cells were isolated directly from naïve C57BL/6 (WT), *Mavs^−/−^*, *Ifnar^−/−^*, or DKO splenocytes using the NK cell negative selection Isolation kit (Miltenyi Biotec), according to the manufacturer's instructions (2 rounds of purification; greater than 85% purity). Bone-marrow derived DC were generated as described previously [8]. Briefly, bone marrow cells from WT, *Mavs^−/−^*, *Ifnar^−/−^*, or DKO mice were isolated and cultured for 7 days in RPMI-1640 supplemented with granulocyte-macrophage-colony stimulating factor (20 ng/ml) to generate DC. On day 7, cells were collected and analyzed for CD11b^+^ and CD11c^+^ expression and used when the purity was greater than 90%. 1×10^5^ DCs were infected with WNV-TX at an MOI of 5.0 for 2 hours, washed with serum-free DMEM, resuspended in cRPMI, and cocultured with purified NK cells at a ratio of 1∶2 in a 96-well U-bottom plate (200 µl total volume). After 18 hours, cells were removed from the cultures, stained as described above, and analyzed by flow cytometry.

### Statistical analysis

Kaplan-Meier survival curves were analyzed by the log-rank test. For *in vivo* viral burden analysis, Mann-Whitney analysis was used to determine statistical differences. For immune cell analysis, an unpaired Student's t-test was used to determine statistical differences. A *P*≤0.05 was considered statistically significant. All data were analyzed using Prism software (GraphPad Prism5).

## Supporting Information

Table S1 **Liver genes regulated by the RLR and type I IFN signaling pathways.** Venn analysis was performed between differentially expressed genes from WT and KO infected livers to identify RLR-dependent (WT compared to *Mavs^−/−^*; 360 genes), type I IFN-dependent (WT compared to *Ifnar^−/−^*; 282 genes), and RLR- and type I IFN-dependent (WT compared DKO; 464 genes) genes.
(PDF)

Table S2 **WT infected spleen top IPA canonical pathways.** Top scoring canonical pathways enriched from WT infected spleens (1124 differentially expressed genes).
(PDF)

Table S3 **WT infected liver top IPA canonical pathways.** Top scoring canonical pathways enriched from WT infected livers (658 differentially expressed genes).
(PDF)

Table S4 **Canonical pathways annotated in cytoscape enrichment map.** Key for node identification in Figure 5. Table is organized by biological function followed by the node number, associated canonical pathway, and B-H *P* value. Canonical pathways depicted in italics are enriched in both WT infected spleens and livers.
(PDF)
