# Supplementary material for: A Systems Biology Approach Reveals that Tissue Tropism to West Nile Virus Is Regulated by Antiviral Genes and Innate Immune Cellular Processes
Source: PLoS Pathog. 2013 Feb 7;9(2):e1003168. doi: 10.1371/journal.ppat.1003168 (PMC3567171; doi:10.1371/journal.ppat.1003168)
Supplement: Table S1 — Liver genes regulated by the RLR and type I IFN signaling pathways. Venn analysis was performed between differentially expressed genes from WT and KO infected livers to identify RLR-dependent (WT compared to Mavs−/−; 360 genes), type I IFN-dependent (WT compared to Ifnar−/−; 282 genes), and RLR- and type I IFN-dependent (WT compared DKO; 464 genes) genes. (PDF) [file ppat.1003168.s001.pdf]

Table S1: Liver genes regulated by the RLR and Type I IFN signaling pathways

RLR-dependent	Type I IFN-dependent	RLR- and Type I IFN-dependent
1110032L06Rik	1110032L06Rik	1110032L06Rik
1190002F15Rik	1190002F15Rik	1190002F15Rik
1600017P15Rik	1600017P15Rik	1600017P15Rik
1700007K13Rik	1700026L06Rik	1700007K13Rik
1700025G04Rik	1700029J07Rik	1700025G04Rik
1700029I01Rik	2010317E24Rik	1700026L06Rik
1700029J07Rik	2310002F09Rik	1700029I01Rik
1810012P15Rik	2810408P10Rik	1700029J07Rik
1810013L24Rik	2810416G20Rik	1810013L24Rik
2010111I01Rik	2810417H13Rik	2310002F09Rik
2010317E24Rik	2810454L23Rik	2810408P10Rik
2310002F09Rik	2900072G19Rik	2810417H13Rik
2810408P10Rik	3110082D06Rik	2900009J20Rik
2810416G20Rik	3830403N18Rik	2900072G19Rik
2810417H13Rik	4930415C11Rik	3110082D06Rik
2810454L23Rik	4930434E21Rik	3830403N18Rik
2900009J20Rik	4930535E02Rik	4930415C11Rik
2900072G19Rik	4930562F07Rik	4930434E21Rik
3830403N18Rik	4930567H12Rik	4930486L24Rik
4633401B06Rik	4933408J17Rik	4930524L23Rik
4833417J20Rik	5730488B01Rik	4930535E02Rik
4930415C11Rik	6430500C12Rik	4930562F07Rik
4930434E21Rik	6530418L21Rik	4930567H12Rik
4930524L23Rik	8430419K02Rik	4933408J17Rik
4930535E02Rik	9330174C13Rik	5430427O19Rik
4930562F07Rik	9430083B18Rik	5430435G22Rik
4930567H12Rik	9530083O12Rik	5730488B01Rik
4933408J17Rik	9630055L06Rik	6430500C12Rik
5430435G22Rik	A530023O14Rik	6530418L21Rik
5730488B01Rik	Abi3	6720475J19Rik
6430500C12Rik	Acap1	9330174C13Rik
6530418L21Rik	Acot6	9430083B18Rik
6720475J19Rik	Adam12	9530028C05
8430419K02Rik	Adcy7	9530083O12Rik
9330174C13Rik	Adrb1	9630055L06Rik
9430083B18Rik	AI504432	A430084P05Rik
9530028C05	Alk	A530032D15Rik
9530083O12Rik	Amy1	Abcg1
9630055L06Rik	Arhgap15	Abhd8
A530023O14Rik	Arhgap36	Abi3
Abcg1	Arhgap9	Acap1
Abhd8	Asb2	Acot6
Abi3	AV039307	Acvrl1
Acvrl1	AV249152	Adam12
Adam12	B3galnt1	Adcy1
Adam23	B930095I24Rik	Adcy7
Adcy1	BB431852	Adrb1
Adcy7	BC055324	AF251705
Adrb1	Birc5	Agtrap
AF251705	Btbd16	AI504432
Agtrap	Bub1b	Aif1
AI504432	C1qa	Aldh3b1
Alk	C1qb	Alk
Amy1	C1qc	Amy1
Arhgap36	C230052I12Rik	Arhgap15

RLR-dependent	Type I IFN-dependent	RLR- and Type I IFN-dependent
Arhgap9	C5ar1	Arhgap25
Armc7	Cacna1c	Arhgap30
Asb2	Camk2b	Arhgap36
Aurka	Card11	Arhgap9
Aurkb	Cbr2	Armc7
AV039307	Ccdc106	AV039307
AV249152	Ccdc67	AV249152
B3galnt1	Ccna2	Axl
B430306N03Rik	Ccnb1	B3galnt1
B930095I24Rik	Ccnb2	B430306N03Rik
BB431852	Ccr5	B930095I24Rik
BC023105	Ccr8	Batf3
BC055324	Cd180	BB431852
Bcl2a1b	Cd200r1	BC013712
Birc5	Cd300ld	BC023105
Btbd16	Cd5l	BC055324
Bub1b	Cd72	Bcl2a1b
C130026I21Rik	Cd84	Bst2
C1qb	Cdc25b	Btbd16
C1qtnf3	Cdca5	Btk
C230052I12Rik	Cdca8	Bub1b
C5ar1	Cdk1	C130026I21Rik
Cacna1c	Cenpe	C1qa
Camk2b	Chrnb2	C1qb
Casp4	Ckap2l	C1qc
Cbr2	Ckb	C1qtnf3
Ccdc106	Clec10a	C230052I12Rik
Ccdc67	Clec1b	C5ar1
Ccl2	Clec4a2	Cacna1c
Ccl3	Clec4a3	Camk2b
Ccl4	Clec4b1	Card11
Ccl6	Clec7a	Casp1
Ccna2	Clspn	Ccdc106
Ccnb1	Col13a1	Ccdc67
Ccnb2	Crtc3	Ccdc88b
Ccr8	Cryba4	Ccr5
Cd180	Cxcl13	Ccr8
Cd200r1	Cysltr2	Cd180
Cd300a	Cyth4	Cd200r1
Cd300ld	D730047E02Rik	Cd300a
Cd5l	Dbf4	Cd300e
Cdc25b	Dck	Cd300ld
Cdc7	Defb1	Cd44
Cdca2	Depdc1a	Cd48
Cdca5	Dlgap5	Cd52
Cdca8	Dnahc5	Cd53
Cdk1	Dock10	Cd5l
Cdkn3	Dppa3	Cd84
Cenpe	Dscc1	Cd86
Cenpi	Dse	Cdkn3
Cerk	Dync2h1	Cds1
Cfp	E030037K03Rik	Cfp
Chrnb2	E2f2	Chit1
Ciita	Efcab3	Chrnb2
Ckap2l	Emr1	Ciita
Ckb	Emr4	Clec10a
Clec10a	Epsti1	Clec1b

RLR-dependent	Type I IFN-dependent	RLR- and Type I IFN-dependent
Clec4f	Etaa1	Clec4a2
Clspn	Evi2a	Clec4a3
Col13a1	Exosc7	Clec4b1
Col4a1	Expi	Clec7a
Cpt1a	Fam105a	Cmpk2
Crtc3	Fancd2	Col13a1
Cryba4	Fanci	Crtc3
Cx3cr1	Fbxo45	Cryba4
Cxcl13	Fgd2	Ctla2b
Cyp4a10	Filip1l	Ctss
Cysltr2	Fmr1nb	Cxcl10
D17H6S56E-5	Frmd4a	Cxcl13
D730047E02Rik	Frmd5	Cxcl9
Dbf4	Fut7	Cybb
Dck	Gabra2	Cysltr2
Defb1	Galnt6	Cyth4
Depdc1a	Gbgt1	D14Ertd668e
Dnahc5	Gins2	D17H6S56E-5
Dock10	Gja6	D730047E02Rik
Dppa3	Glipr1	Dck
Dscc1	Gm14025	Ddx58
Dse	Gm3740	Defb1
Dusp8	Gm4870	Dhx58
Dync2h1	Gm4907	Dlgap5
E2f2	Gm8995	Dock10
Edn1	Gnaz	Dock2
Efcab3	Gprasp1	Dok2
Eif2ak3	Gtse1	Dppa3
Eml6	Gzma	Dse
Emr4	Hist3h2ba	Dync2h1
Enox1	Hpgds	E030037K03Rik
Epb4.1l3	Hrh1	Ear1
Ero1lb	Hsh2d	Ear10
Etaa1	Il10	Ebi3
Exosc5	Incenp	Edn1
Exosc7	Iqch	Efcab3
Expi	Kcna3	Eif2ak2
Fabp7	Kif14	Emr1
Fam105a	Kif18b	Emr4
Fam55d	Kif20a	Enox1
Fanci	Kif20b	Entpd1
Fastkd1	Kif2c	Epb4.1l3
Fbxl17	Kif4	Epsti1
Fbxo45	Kiss1	Ero1lb
Filip1l	Klra12	Etaa1
Fmr1nb	Klra15	Evi2a
Folr2	Klra16	Evl
Frmd4a	Klra23	Exosc5
Frmd5	Klra7	Exosc7
Fut7	Klrk1	Expi
Fzd5	Krt85	Fabp7
G6pdx	Lamp3	Fam105a
Gabra2	Lancl3	Fam129a
Galnt6	Laptm5	Fanci
Gbgt1	Lce1e	Fbxo45
Gbp6	Lgr4	Fbxw17
Ggnbp1	Lilra5	Fcer1g

RLR-dependent	Type I IFN-dependent	RLR- and Type I IFN-dependent
Gins2	LOC100045212	Fcgr1
Gja6	Lpxn	Fcgr4
Glipr1	Lrrc25	Fermt3
Gm11428	Lysmd4	Fgd2
Gm11545	Mchr1	Fgl2
Gm13157	Mep1b	Filip1l
Gm14025	Mill2	Fmr1nb
Gm3740	Minpp1	Folr2
Gm4870	Mlf1ip	Fpr1
Gm4907	Mrc2	Frmd4a
Gm5933	Mtap2	Frmd5
Gm6484	Mto1	Fut7
Gm71	Mx1	Fyb
Gm8995	Mxd3	Fzd5
Gnaz	Mybpc2	G6pdx
Gpr35	Myo1a	Gabra2
Grin2b	Naip5	Galnt6
Gtse1	Ncapg	Gbgt1
Havcr2	Ncapg2	Gbp2
Hist3h2ba	Ncr1	Gbp3
Hpgds	Ndc80	Gbp5
Hrh1	Neil3	Gbp6
Hsh2d	Nell1	Gbp8
Ifi203	Nrg3	Gbp9
Ifi27l2b	Nusap1	Ggnbp1
Il10	Olfr1136	Gja6
Il10ra	Olfr1294	Glipr1
Iqch	Olfr1377	Gm11428
Irak3	Olfr1462	Gm11545
Kcna3	Olfr365	Gm12250
Kif12	Olfr466	Gm14025
Kif14	Olfr560	Gm14446
Kif18b	Olfr591	Gm1966
Kif20a	Olfr888	Gm2397
Kif20b	Olfr934	Gm3740
Kif22	Onecut3	Gm4907
Kif2c	Pabpc1	Gm5431
Kif4	Panx3	Gm5933
Kiss1	Pask	Gm6484
Klra15	Pbk	Gm7035
Krt85	Pcdhb17	Gm71
Lamp3	Pdlim4	Gm8995
Lancl3	Pira6	Gng2
Lce1e	Pitx1	Gngt2
Lcn2	Pkd1l2	Gprasp1
Lgmn	Pld4	Gtpbp10
Lgr4	Plk1	Gtse1
Lhx1	Plk4	Gvin1
Lilrb3	Pou3f1	Gzma
Lilrb4	Ppfia4	Gzmb
Lpcat2	Ppp1r14c	H2-M3
Lpxn	Ppp1r1a	H2-Q8
Lrrc25	Pram1	H2-T22
Lysmd4	Prex2	H2-T24
Mchr1	Prl2c5	Hck
Mcoln2	Prss52	Hcls1
Med12l	Psg22	Herc5

RLR-dependent	Type I IFN-dependent	RLR- and Type I IFN-dependent
Mep1b	Psrc1	Hk3
Mill2	Ptgdr	Hpgds
Minpp1	Ptgs1	Hrh1
Mlf1ip	Ptprr	Hsh2d
Mll2	Rab19	I830012O16Rik
Mrc2	Rab39	Ifi204
Ms4a7	Racgap1	Ifi205
Mtap2	Rad54b	Ifi27l1
Mto1	Rel	Ifi27l2b
Mxd3	Rgs14	Ifi30
Mybpc2	Rgs2	Ifi44
Myo1a	Rhoh	Ifi47
Naip5	Rnd3	Ifit1
Ncapg	Ryr3	Ifit2
Ncapg2	Sash1	Ifitm7
Ndc80	Sash3	Il10
Neil3	Sat1	Il10ra
Nell1	Scg5	Il18bp
Niacr1	Sfpi1	Il2rg
Nrg3	Sgol2	Iqch
Nudt6	Sidt1	Irf7
Nusap1	Siglec1	Irf9
Oas1b	Skint9	Irgm1
Olfr1136	Slc37a2	Irgm2
Olfr1294	Slc5a11	Isg15
Olfr1377	Slc6a18	Itgal
Olfr1462	Smc1b	Kcna3
Olfr365	Sp100	Kif12
Olfr466	Spag16	Kif18b
Olfr560	Spnb1	Kif2c
Olfr591	Sprr2k	Kiss1
Olfr888	Stat5a	Klra12
Olfr934	Sv2a	Klra15
Onecut3	Svs3a	Klra16
Pabpc1	Syt13	Klra2
Panx3	Tacc3	Klra23
Pbk	Tbxas1	Klra7
Pcdhb17	Tcstv1	Klrk1
Pdk4	Tlr7	Krt85
Pdlim4	Tmem229a	Lair1
Pilra	Tmem229b	Lamp3
Pilrb1	Tmem26	Lce1e
Pilrb2	Tmem86a	Lcp2
Pira2	Tnfrsf11a	Lgals3bp
Pira6	Tpx2	Lgr4
Pitx1	Ubc	Lilra5
Pkd1l2	Ube2c	Lilrb4
Pkib	Vmn1r193	LOC100045212
Plekha2	Vmn1r5	Lpcat2
Plk1	Wdfy4	Lpxn
Plk4	Xcr1	Lrrc25
Ppap2b	Xlr	Lst1
Ppp1r14c	Zfp516	Ly6a
Ppp1r1a	Zfp711	Ly6e
Prex2	Zfp862	Ly86
Prl2c5		Ly9
Prss52		Lysmd4

RLR-dependent	RLR- and Type I IFN-dependent
Psg22	March1
Psrc1	Marco
Ptgdr	Mchr1
Ptplad2	Mcoln2
Ptpn22	Med12l
Ptprr	Mep1b
Pyhin1	Minpp1
Rab37	Mlf1ip
Racgap1	Mll2
Rad54b	Mnda
Rapgef2	Mpa2l
Rcan3	Mpeg1
Rel	Mrc2
Rgs1	Ms4a4b
Rhpn2	Ms4a4c
Rinl	Ms4a4d
Rnd3	Ms4a6b
Ryr3	Ms4a6c
Samsn1	Ms4a6d
Sash1	Ms4a7
Sat1	Msr1
Scg5	Mtap2
Sgol2	Mx1
Sidt1	Mx2
Sirpa	Mybpc2
Skint9	Myo1a
Sla	Naip5
Slc15a3	Nckap1l
Slc37a2	Ncoa7
Slc40a1	Ncr1
Slc6a18	Ndc80
Slc7a8	Nell1
Spag16	Nkg7
Spnb1	Nrg3
Sprr2k	Nudt6
Stat5a	Oas1a
Sv2a	Oas1b
Svs3a	Oas1f
Sybu	Oas3
Syt13	Oasl1
Tacc3	Oasl2
Tbxas1	Olfr1136
Tcstv1	Olfr1294
Tfg	Olfr1377
Timd4	Olfr1462
Tlr2	Olfr365
Tlr3	Olfr466
Tlr6	Olfr560
Tmem229a	Olfr591
Tmem229b	Olfr888
Tmem26	Olfr934
Tmem44	Onecut3
Tmem67	P2ry13
Tmem86a	Pabpc1
Tmsb10	Panx3
Tnf	Pard3b
Top2a	Pbk

RLR-dependent

Tpx2
Treml4
Trim68
Trpv2
Tspyl5
Tyrobp
Ubc
Ube2c
Uck1
Vasp
Vmn1r193
Vmn1r5
Vti1a
Was
Xlr
Zfp516
Zfp711
Zfp862
Zfp9

RLR- and Type I IFN-dependent

Pdk4
Pdlim4
Phf11
Pik3cd
Pilra
Pilrb1
Pilrb2
Pira6
Pitx1
Pkd1l2
Pkib
Pla2g4a
Pld4
Plekha2
Plk4
Pou3f1
Ppap2b
Ppfia4
Ppp1r1a
Prex2
Prl2c5
Prss52
Psg22
Psmb8
Ptgdr
Ptplad2
Ptprc
Ptprr
Pyhin1
Rab19
Rab37
Rab39
Rac2
Racgap1
Rad54b
Rcan3
Rel
Rgs2
Rhoh
Rhpn2
Rinl
Rnd3
Rtp4
Ryr3
Sash1
Serpina3g
Sfpi1
Sgcb
Shisa5
Sidt1
Siglec1
Sirpa
Skint9
Slamf9
Slc11a1
Slc15a3
Slc37a2

RLR- and Type I IFN-dependent
Slc40a1
Slc5a11
Slc6a18
Slc7a8
Slfn1
Slfn8
Slfn9
Smc1b
Soat1
Sp100
Sp110
Spag16
Spic
Spnb1
Stat1
Stat2
Stat5a
Stox2
Sv2a
Svs3a
Sybu
Syt13
Tap1
Tbxas1
Tcfec
Tcstv1
Tfg
Tgtp1
Timd4
Tlr1
Tlr2
Tlr3
Tlr7
Tmem229a
Tmem229b
Tmem26
Tmem67
Tmem86a
Tmsb4x
Tnfrsf11a
Tnfrsf14
Tor3a
Treml4
Trex1
Trim30
Trim68
Trim79
Tspyl5
Tyrobp
Ubc
Ube2c
Ube2l6
Uck1
Usp18
Vav1
Vcam1
Vmn1r193

RLR- and Type I IFN-dependent
Vmn1r5
Was
Wipf1
Xaf1
Xcr1
Xlr
Zbp1
Zfp516
Zfp711
Znfx1
