# Supplementary material for: A Systems Biology Approach Reveals that Tissue Tropism to West Nile Virus Is Regulated by Antiviral Genes and Innate Immune Cellular Processes
Source: PLoS Pathog. 2013 Feb 7;9(2):e1003168. doi: 10.1371/journal.ppat.1003168 (PMC3567171; doi:10.1371/journal.ppat.1003168)
Supplement: Table S2 — WT infected spleen top IPA canonical pathways. Top scoring canonical pathways enriched from WT infected spleens (1124 differentially expressed genes). (PDF) [file ppat.1003168.s002.pdf]

Table S2: WT infected spleen top IPA canonical pathways

Ingenuity Canonical Pathway	B-H *P* value
Interferon Signaling	2.00E-12
Role of Hypercytokinemia/hyperchemokinemia in the Pathogenesis of Influenza	1.15E-07
Activation of IRF by Cytosolic Pattern Recognition Receptors	1.20E-07
Communication between Innate and Adaptive Immune Cells	2.29E-06
Role of BRCA1 in DNA Damage Response	2.29E-06
Role of Pattern Recognition Receptors in Recognition of Bacteria and Viruses	2.29E-06
Crosstalk between Dendritic Cells and Natural Killer Cells	7.94E-06
Antigen Presentation Pathway	7.94E-06
Pyrimidine Metabolism	3.16E-05
Role of CHK Proteins in Cell Cycle Checkpoint Control	4.07E-05
Allograft Rejection Signaling	4.07E-05
Pathogenesis of Multiple Sclerosis	6.61E-05
Graft-versus-Host Disease Signaling	9.33E-05
Role of RIG-I-like Receptors in Antiviral Innate Immunity	1.41E-04
Retinoic acid Mediated Apoptosis Signaling	3.09E-04
Cytotoxic T Lymphocyte-mediated Apoptosis of Target Cells	4.07E-04
Protein Ubiquitination Pathway	4.07E-04
Neuroprotective Role of THOP1 in Alzheimer's Disease	4.79E-04
Role of JAK1, JAK2 and TYK2 in Interferon Signaling	1.32E-03
Cell Cycle: G2/M DNA Damage Checkpoint Regulation	1.38E-03
Systemic Lupus Erythematosus Signaling	2.04E-03
Mitotic Roles of Polo-Like Kinase	2.04E-03
Role of Lipids/Lipid Rafts in the Pathogenesis of Influenza	2.04E-03
Mismatch Repair in Eukaryotes	2.04E-03
Cell Cycle Control of Chromosomal Replication	2.75E-03
Type I Diabetes Mellitus Signaling	1.00E-02
Autoimmune Thyroid Disease Signaling	1.17E-02
Role of Cytokines in Mediating Communication between Immune Cells	1.55E-02
Hereditary Breast Cancer Signaling	1.66E-02
ATM Signaling	1.66E-02
Role of PI3K/AKT Signaling in the Pathogenesis of Influenza	1.66E-02
OX40 Signaling Pathway	1.78E-02
Role of PKR in Interferon Induction and Antiviral Response	2.00E-02
One Carbon Pool by Folate	2.09E-02
Cell Cycle: G1/S Checkpoint Regulation	2.29E-02
Differential Regulation of Cytokine Production in Intestinal Epithelial Cells by IL-17A and IL-17F	3.02E-02
p53 Signaling	3.02E-02
Cyclins and Cell Cycle Regulation	3.72E-02
Role of JAK1 and JAK3 in γc Cytokine Signaling	3.72E-02
Dendritic Cell Maturation	5.25E-02
Role of IL-17F in Allergic Inflammatory Airway Diseases	7.24E-02
IL-15 Production	7.24E-02
Atherosclerosis Signaling	8.32E-02
