# Supplementary material for: A Systems Biology Approach Reveals that Tissue Tropism to West Nile Virus Is Regulated by Antiviral Genes and Innate Immune Cellular Processes
Source: PLoS Pathog. 2013 Feb 7;9(2):e1003168. doi: 10.1371/journal.ppat.1003168 (PMC3567171; doi:10.1371/journal.ppat.1003168)
Supplement: Table S3 — WT infected liver top IPA canonical pathways. Top scoring canonical pathways enriched from WT infected livers (658 differentially expressed genes). (PDF) [file ppat.1003168.s003.pdf]

Table S3: WT infected liver top IPA canonical pathways

Ingenuity Canonical Pathway	B-H *P* value
Role of Pattern Recognition Receptors in Recognition of Bacteria and Viruses	3.16E-14
Activation of IRF by Cytosolic Pattern Recognition Receptors	1.70E-07
TREM1 Signaling	6.17E-07
Communication between Innate and Adaptive Immune Cells	2.14E-06
Natural Killer Cell Signaling	3.39E-06
Interferon Signaling	3.89E-06
Allograft Rejection Signaling	1.07E-04
Crosstalk between Dendritic Cells and Natural Killer Cells	2.40E-04
Leukocyte Extravasation Signaling	5.50E-04
Pathogenesis of Multiple Sclerosis	1.26E-03
Fcγ Receptor-mediated Phagocytosis in Macrophages and Monocytes	1.26E-03
Altered T Cell and B Cell Signaling in Rheumatoid Arthritis	1.26E-03
Toll-like Receptor Signaling	8.91E-03
T Helper Cell Differentiation	8.91E-03
Dendritic Cell Maturation	8.91E-03
Fc Epsilon RI Signaling	9.33E-03
iCOS-iCOSL Signaling in T Helper Cells	9.33E-03
NF-κB Signaling	1.02E-02
Systemic Lupus Erythematosus Signaling	1.07E-02
Mitotic Roles of Polo-Like Kinase	1.74E-02
Cell Cycle: G2/M DNA Damage Checkpoint Regulation	1.74E-02
Cytotoxic T Lymphocyte-mediated Apoptosis of Target Cells	2.40E-02
Differential Regulation of Cytokine Production in Intestinal Epithelial Cells by IL-17A and IL-17F	2.40E-02
Autoimmune Thyroid Disease Signaling	3.39E-02
PI3K Signaling in B Lymphocytes	3.39E-02
Graft-versus-Host Disease Signaling	4.57E-02
PKCθ Signaling in T Lymphocytes	4.57E-02
Primary Immunodeficiency Signaling	4.57E-02
Production of Nitric Oxide and Reactive Oxygen Species in Macrophages	4.57E-02
Role of BRCA1 in DNA Damage Response	4.79E-02
Role of PKR in Interferon Induction and Antiviral Response	4.79E-02
Eicosanoid Signaling	5.01E-02
Colorectal Cancer Metastasis Signaling	5.62E-02
Role of Hypercytokinemia/hyperchemokinemia in the Pathogenesis of Influenza	6.17E-02
Cdc42 Signaling	8.13E-02
Sphingosine-1-phosphate Signaling	8.13E-02
Differential Regulation of Cytokine Production in Macrophages and T Helper Cells by IL-17A and IL-17F	8.13E-02
Role of RIG-I-like Receptors in Antiviral Innate Immunity	8.13E-02
CD28 Signaling in T Helper Cells	1.00E-01
Chemokine Signaling	1.01E-01
ATM Signaling	1.02E-01
IL-9 Signaling	1.02E-01
