# Supplementary material for: A Systems Biology Approach Reveals that Tissue Tropism to West Nile Virus Is Regulated by Antiviral Genes and Innate Immune Cellular Processes
Source: PLoS Pathog. 2013 Feb 7;9(2):e1003168. doi: 10.1371/journal.ppat.1003168 (PMC3567171; doi:10.1371/journal.ppat.1003168)
Supplement: Table S4 — Canonical pathways annotated in cytoscape enrichment map. Key for node identification in Figure 5. Table is organized by biological function followed by the node number, associated canonical pathway, and B-H P value. Canonical pathways depicted in italics are enriched in both WT infected spleens and livers. (PDF) [file ppat.1003168.s004.pdf]

Table S4: Canonical pathways annotated in cytoscape enrichment map

No.	Type I Interferon signaling	B-H *P* value
1	*Role of Pattern Recognition Receptors in Recognition of Bacteria and Viruses*	3.16E-14
2	*Activation of IRF by Cytosolic Pattern Recognition Receptors*	1.70E-07
3	*Interferon Signaling*	3.89E-06
4	Toll-like Receptor Signaling	8.91E-03
5	*Role of PKR in Interferon Induction and Antiviral Response*	4.79E-02
6	*Role of RIG-I-like Receptors in Antiviral Innate Immunity*	8.13E-02

No.	Innate cellular immune response	B-H *P* value
1	*Communication between Innate and Adaptive Immune Cells*	2.14E-06
2	Natural Killer Cell Signaling	3.39E-06
3	*Crosstalk between Dendritic Cells and Natural Killer Cells*	2.40E-04
4	Fcγ Receptor-mediated Phagocytosis in Macrophages and Monocytes	1.26E-03
5	Dendritic Cell Maturation	8.91E-03
6	Production of Nitric Oxide and Reactive Oxygen Species in Macrophages	4.57E-02

No.	Adaptive cellular immune response	B-H *P* value
1	*Allograft Rejection Signaling*	1.07E-04
2	Leukocyte Extravasation Signaling	5.50E-04
3	T Helper Cell Differentiation	8.91E-03
4	iCOS-iCOSL Signaling in T Helper Cells	9.33E-03
5	*Cytotoxic T Lymphocyte-mediated Apoptosis of Target Cells*	2.40E-02
6	Graft-versus-Host Disease Signaling	4.57E-02
7	PKCθ Signaling in T Lymphocytes	4.57E-02
8	CD28 Signaling in T Helper Cells	1.00E-01

No.	Disease-specific signaling	B-H *P* value
1	*Pathogenesis of Multiple Sclerosis*	1.26E-03
2	Altered T Cell and B Cell Signaling in Rheumatoid Arthritis	1.26E-03
3	Systemic Lupus Erythematosus Signaling	1.07E-02
4	Autoimmune Thyroid Disease Signaling	3.39E-02
5	Primary Immunodeficiency Signaling	4.57E-02
6	Colorectal Cancer Metastasis Signaling	5.62E-02
7	*Role of Hypercytokinemia/hyperchemokinemia in the Pathogenesis of flu*	6.17E-02

No.	Cytokine Signaling	B-H *P* value
1	TREM1 Signaling	6.17E-07
2	NF-κB Signaling	1.02E-02
3	Diff. Regulation of Cytokines in Intestinal Epithelial Cells by IL-17A and IL-17F	2.40E-02
4	Diff. Regulation of Cytokine in Macrophages and Th Cells by IL-17A and IL-17F	8.00E-02

No.	Cell Stress and Injury	B-H *P* value
1	*Cell Cycle: G2/M DNA Damage Checkpoint Regulation*	1.74E-02
2	*Mitotic Roles of Polo-Like Kinase*	1.74E-02

No.	Humoral immune response	B-H *P* value
1	Fc Epsilon RI Signaling	9.33E-03
2	PI3K Signaling in B Lymphocytes	3.39E-02

No.	Cell Growth	B-H *P* value
1	Role of BRCA1 in DNA Damage Response	4.79E-02
2	Cdc42 Signaling	8.13E-02

No.	Intracellular secondary messenger signaling	B-H *P* value
1	Sphingosine-1-phosphate Signaling	8.13E-02
